# Measuring Social Inclusion in Europe: a non-additive approach with the expert-preferences of public policy planners

**DOI:** 10.1093/jrsssa/qnad106

**Published:** 2023-09-05

**Authors:** Ludovico Carrino, Luca Farnia, Silvio Giove

**Affiliations:** Department of Global Health and Social Medicine, King’s College London, London, UK; Department of Economics, Management, Mathematics and Statistics "Bruno de Finetti" (DEAMS), University of Trieste, Trieste, Italy; Fondazione Eni Enrico Mattei, Venezia, Italy; Department of Economics, Ca’ Foscari University, Venezia, Italy

**Keywords:** European regions, aggregation, Choquet integral, non-additive measures, normalisation strategy, normative weights, scenario evaluation, social exclusion, social inclusion

## Abstract

This paper introduces a normative, expert-informed, time-dependent index of Social Inclusion for European administrative regions in five countries, using longitudinal data from Eurostat. Our contribution is twofold: first, our indicator is based on a non-additive aggregation operator (the Choquet Integral), which allows us to model many preferences’ structures and to overcome the limitations embedded in other approaches. Second, we elicit the parameters of the aggregation operator from an expert panel of Italian policymakers in Social Policy, and Economics scholars. Our results highlight that Mediterranean countries exhibit lower Inclusion levels than Northern/Central countries, and that this disparity has grown in the last decade. Our results complement and partially challenge existing evidence from data-driven aggregation methods.

## 1. Introduction

In the last two decades, economists have developed a growing interest in multidimensional measures of complex social phenomena, such as poverty and well-being (Stiglitz et al., 2010), as a tool to evaluate the outcomes of public policies and the Welfare State (Atkinson 2005; Pestieau & Lefebvre 2018). In the European context, an extensive focus by policymakers and academics has been devoted to Social Inclusion, conceptualised as an enlarged measure of poverty that accounts for the interlinks between income, education, health, and labour market conditions. As Social Inclusion features prominently within the European Union’s agenda for social integration and development, e.g. Europe 2010, 2020, and 2030 Strategies (Atkinson et al., 2002; Vanhercke 2012), policymakers require tools to monitor its progress across country and time (Atkinson 2005; Rogge & Konttinen 2018; Stiglitz et al., 2010). A few studies have proposed a specific composite measure of Social Inclusion, i.e. through the aggregation of observed performances from several sub-components (attributes) into an index. However, their results are mostly descriptive in that they do not aim to provide a normative evaluation of the observed levels of Inclusion across countries. This paper introduces a rigorous approach to the construction of a normative-based index of Social Inclusion, using European regional longitudinal data from Eurostat.

Sen & Anand (1997) highlight how there are inescapable elements of subjectivity (judgement) in any stage of creating a multidimensional index. Social Inclusion is no exception. Creating a Social Inclusion index requires facing two main empirical challenges, which call for subjective and arbitrary methodological choices (Decancq & Lugo 2013; Sen & Anand 1997) that may affect both the results and the interpretation of the results (see, e.g. the theoretical discussions by Alkire et al., (2015); Bosmans et al., (2015), Bourguignon & Chakravarty (2003); Decancq & Lugo (2013); Klugman et al., (2011), Maggino (2017); Ravallion (2011); Stiglitz et al., (2010); Tsui (2002); and the empirical discussions by Carrino (2017); Cavapozzi et al., (2015); D’Ambrosio et al., (2011); Decancq et al., (2019); Deutsch & Silber (2005); Döpke et al., (2017); Ravallion (2012); Saisana et al., (2005); Cohen & Saisana (2014)).

First, it requires to choose a suitable aggregation function that models the relationship between attributes of Social Inclusion. Several studies in poverty measurement have acknowledged that a linear aggregation model embeds a major shortcoming, in that it assumes perfect substitutability among sub-components (i.e. it assumes that the ‘whole’ is equal the ‘sum of its parts’), and have therefore proposed nonlinear aggregation models. However, to our knowledge, no study of Social Inclusion has yet exploited a nonlinear algorithm to characterise the degree of complementarity or substitutability for each combination of sub-components.

Second, creating a multidimensional index requires to estimate the parameters of the aggregation function (e.g. the weights). Such estimation can follow *normative* methods, e.g. eliciting decision-makers’ preferences, or *positive* methods, e.g. with data-driven approaches (Decancq & Lugo 2013). Most recent studies on Social Inclusion in Europe adopt data-driven approaches, e.g. Benefit of the Doubt (BOD), Principal Component Analysis, Factor Analysis, where weights are determined by the statistical properties of the available data (see Giambona and & Vassallo (2014); Lefebvre et al., (2010); Rogge & Self (2019) and Rogge & Konttinen (2018) ). While being widely adopted, valid and relevant, data-driven methods have two main limitations (Decancq & Lugo 2013). First, as its parameters lack an explicit value judgement, a data-driven index should be interpreted under a *positive,* also called *descriptive* perspective. Such an index would, for example, allow to rank countries depending on their Social Inclusion score. While this evaluation can prove highly informative, it might fail to recognise that the first- and last-ranked countries would not necessarily represent socially desirable or undesirable condition. Second, data-driven parameters have a limited economic interpretation. For example, within the BOD framework, many attributes of an index are often assigned (quasi) zero weight. While this is not an ‘unfair’ choice from a statistical perspective (e.g. Rogge & Konttinen (2018)), this allocation is not conceived to reflect an explicit social preference. Hence, data-driven indices allow to make statements about facts, while they are not ideal to draw normative policy recommendations, i.e. statement about values (Decancq & Lugo 2013). Conversely, when the parameters are elicited through normative methods (e.g. experts-elicitation), the index has a *normative* interpretation.

This paper introduces a novel normative and non-additive index of Social Inclusion for European regions in Belgium, Denmark, Germany, Italy and Spain between 2004 and 2017. Our conceptual and operational definition of Social Inclusion is borrowed by the work of the Atkinson commission (Atkinson et al., 2002) which, since the European Council of Laeken in 2001, has been one of the cornerstones of the European initiatives to monitor countries’ progress towards reducing poverty and support the Social Policy Agenda of the EU (UNECE 2022). Such framework has been widely adopted in empirical studies on this topic (e.g. Rogge & Konttinen (2018); Mazziotta & Pareto (2016), Carrino (2016); (Rogge & Konttinen 2018) Lefebvre, Coelli & Pestieau (2010)). Our work offers three important contributions to the literature. First, we use the Choquet Integral as aggregation operator (Grabisch 1996), which allows to overcome the major limitation of the standard linear model, with a more flexible approach than most alternatives employed in the literature of poverty measurement. Indeed, the Choquet Integral allows to estimate both the contribution of each attribute to the overall Social Inclusion, and the degree of complementarity/substitutability for each pair (coalition) of attributes. We argue that such an approach is well suited to measure Social Inclusion, which is characterised by attributes having potentially different pairwise interactions. Nevertheless, while the Choquet Integral has been implemented in studies on well-being, sustainability, and inequality (Angilella et al., 2014; Bertin et al., 2018; Gajdos 2002; Meyer & Ponthière 2011; Pinar et al., 2014), it has not yet been applied to Social Inclusion.

Second, our index is among the first to provide a *normative* index of Social Inclusion, as its weights are elicited from the preferences of decision-makers and academics. To enhance the external validity of our index, we elicited the parameters of the aggregation function from the social preferences of a panel of Experts in Social Policy in Italy. Using the scenario elicitation method (e.g. Benjamin et al., (2014)), we asked decision-makers to evaluate the level of Social Inclusion embedded in a set of fictional societies, as defined by the values of four indicators of Inclusion, selected according to the existing theory. In order to standardise the scenarios, the indicators were normalised ex ante, using a normalisation function based on the stated preferences of 100 scholars in Economics (Carrino 2016).

Third, we provide novel evidence on Social Inclusion exploiting longitudinal regional data. Regions have been increasingly recognised as key development actors (OECD 2018) and involved in outlining the Action Plans on Social Inclusion established by the European Council (Rogge & Konttinen 2018), hence it is crucial to provide a deeper understanding of geographical discrepancies and trends in Social Inclusion within countries. Moreover, we expand upon previous work by Rogge & Self (2019), as we use time series data to illustrate dynamics between 2004 and 2017.

The strength of our approach lies in its flexibility, joint with its normative nature. As such, we believe it is an ideal complement to the existing literature based on data-driven methods. For future research, increasing the sample size of interviewees and their geographic heterogeneity would be beneficial for statistical robustness; this however does not limit the validity of our findings given the nature of expertise of the interviewees and the clear pattern in the results.

Our results, indeed, point to large disparities in Social Inclusion in Europe between Continental countries (higher Inclusion) and Mediterranean countries (lower Inclusion), which were exacerbated after the 2008 economic crisis. This challenges previous findings from recent data-driven studies (Rogge & Konttinen 2018). Finally, we show that regional variation in Social Inclusion is very pronounced in Italy, and low in Denmark and Germany.

The paper is structured as follows: Section 2 introduces the dataset; Section 3 outlines the methods; Section 4 describes the results; sensitivity and comparability analyses; while Section 5 concludes.

## 2. Social inclusion: conceptual framework and data

Promoting Social Inclusion is a priority target in the European Commission’s strategic vision, as exemplified by large policy initiatives such as the Lisbon Strategy, Europe 2020 and Europe 2030. For example, in 2010 the EU countries committed to reduce by at least 20 million the population at risk of social exclusion, defined as the population either below the relative-poverty threshold, or facing severe material deprivation, or living in (quasi-)jobless (i.e. very low work intensity) households (Social Protection Committee 2018). Social Inclusion was broadly introduced in the economics literature in the 1970s to capture situations where individuals are excluded from the ‘mainstream of society’ even if not income-poor (Sen & Anand 1997). The European institutions have later defined Social Inclusion as an enlarged measure of monetary poverty, as it focuses on the ‘multidimensional nature of the mechanisms whereby individuals and groups are excluded from taking part in the social exchanges, from the component practices and rights of social integration and of identity’ (European Communities Commission 1992). Social Inclusion has therefore a very strong policy connotation: it is intended as a multidimensional phenomenon stemming from inadequacies or weaknesses in public services and policies from various areas, which combine and cumulate to affect both people and regions via cumulative and interdependent processes (Atkinson et al., 2002).

### 2.1 The EU’s Social inclusion conceptual framework

This study adopts the European Union’s definition and indicators of Social Inclusion as defined by Atkinson et al. (2002), the so-called ‘Laeken indicators’. The European Union has for a long time aimed at strengthening the role of social policy as a productive factor, and one of the means to achieve this aim has been establishing a process of information exchange that would allow a constant mutual monitoring of Social Inclusion between Member States, called ‘open method of coordination’ (Borrás & Jacobsson 2004). The process of monitoring required reaching an agreement on what should be the conceptual and operational definition of Social Inclusion, which were both defined by the commission led by Tony Atkinson (Atkinson et al., 2002), and presented at the Laeken European Council in 2001. Such definitions have been since then widely applied in economics and statistics works on Social Inclusion, and we refer to it as the ‘EU Social Inclusion’ concept. Basing on the experience of Member States, the commission identified five basic sub-dimensions of Social Inclusion, hereafter also *attributes*:

- Income: material deprivation;
- Labour market: lack of productive role;
- Education: lack of education;
- Health: poor health;
- Housing: poor housing.

These attributes represent the multidimensional phenomenon by which individuals and groups are excluded from taking part in the social exchanges—to quote the aforementioned original definition of Social Inclusion. The Atkinson commission then identified ten *primary* statistical indicators to measure the first four dimensions. The fifth dimension (housing) was not populated with any statistical indicator, hence we could not consider it in our study.^1^ We refer to Atkinson et al. (2002, 2004), as well as to European Commission (2009, 2010) for further details on the rationale and limitations embedded with both the choice of the Social Inclusion attributes and on the measurement of the 10 primary indicators.

In this study, we propose a novel normative methodological method to create a multidimensional index, employing the EU’s Social Inclusion concept as a policy-relevant case study. For completeness, we briefly recall that the literature in economics and policy has developed alternative conceptual frameworks for measuring poverty and, especially, well-being, the latter being characterised by a wider scope than Social Inclusion (see UNECE (2022) for an exhaustive review of approaches to measure Social Inclusion). A prominent example is the United Nations’ Development Program’s (UNDP) Human Development Index (HDI), which includes three main dimensions (health, living standards and education), hence leaving out labour market performances which are included in the EU’s Social Inclusion (Ravallion 2012). A second example is the Multidimensional Poverty Index (MPI) which was introduced to expand the scope of the HDI (Alkire et al., 2015). The MPI comprises three dimensions such as health, education and living standards (which in turn includes cooking fuel, toilet facility, water access, electricity, flooring material, and assets). Another notable example is the well-being index built by the OECD (OECD 2020), which is based on the conceptual framework developed by Stiglitz et al. (2010). The OECD Well-being Framework includes 11 dimensions, which capture material conditions that shape people’s economic options (Income and Wealth; Housing; Work and Job Quality), other quality-of-life factors related to how well people feel (Health; Knowledge and Skills; Environmental Quality; Subjective Well-being; Safety), and to how connected and engaged people are (Work-Life Balance; Social Connections; Civic Engagement). Finally, the European Union has developed a narrower set of indicators to measure Social Exclusion as part of its Europe 2020 Strategy, which is referred to as the ‘at risk of poverty and social exclusion’ (AROPE) indicators (UNECE 2022). The AROPE approach defines an individual as at risk of poverty or social exclusion when at least one of the following conditions hold: (a) equivalent household income below 60% of national median; (b) households with at least four of the following nine issues: i) impossibility to bear unexpected expenses, ii) cannot afford a week holiday, iii) issues with the mortgage, rent, bills; iv) cannot afford a proper meal every two days; v) not able to adequately heat the house; vi) not able to afford a washing machine vii) a colour TV viii) a phone ix) an automobile; (c) living in families whose members aged 18–59 work less than a fifth of their time. The methodological approach of our paper fully applies to the AROPE indicators, and we compare our findings with the AROPE index in the Results section. In this paper, we prefer to employ the Laeken indicators as case study, as the European Union itself recognises that the Laeken indicators ‘encompass a wider range of issues than the AROPE indicator and move beyond a focus solely on economic and labour market aspects of social exclusion’ (UNECE 2022).

### 2.2 Empirical framework

Our empirical framework employs four statistical indicators among the ten identified by Atkinson et al. (2002) to measure the four attributes of the EU Social Inclusion framework, chosen following existing relevant studies in the field (Lefebvre et al., 2010; Mazziotta & Pareto 2016; Rogge & Konttinen 2018):

- poverty-rate (income)
- long-term unemployment rate (labour market)
- early school-leavers rate (education)
- life expectancy at birth, in years (health)

Table 1 provides a brief definition of the four variables. A similar set of variables has been already used in the literature (Lefebvre et al., 2010; Mazziotta & Pareto 2016; Rogge & Konttinen 2018). We argue that these indicators, although not free from limitations, are relevant and valid, as they cover the most relevant concerns of a modern welfare state, and they reflect aspects that are suitable to analyses that want to enlarge the concept of GDP to better measure social welfare (Lefebvre et al., 2010). In particular, let us briefly discuss each chosen attribute:

- The poverty rate indicator (the percentage falling below income thresholds) is one of the most widely used indicators for the risk of poverty. Compared to an absolute measure of income, it minimises the risk of biases from measurement errors, while providing a context-specific and informative measure of financial shortcomings. Nevertheless, the concept of social exclusion is inherently characterised by the realisation that low income may not be, per se, a reliable indicator of social exclusion, which would require to also evaluate the conditions of other resources and needs of individuals.
- Among the latter factors, shortcomings in the labour market are considered crucial determinants of social security and welfare. In particular, the long-term unemployment rate (percentage unemployed for a year or more) is a key predictor of poverty, as it captures non-transitory periods without the monetary and non-monetary benefits of work, which can have long-lasting effects on individuals’ prospects.
- A similar rationale justifies the choice of education (or lack thereof) as a criterion, and in particular the proportion of early school leavers (individuals aged 18–24 having achieved lower secondary education or less, and not currently attending education or training). Education not only enhances people's productivity at work, but also develops the capacity of individuals to lead a full life, transmitting societal norms and values. In this respect, the indicator measures low educational attainments, which have important influences in subsequent life-chances and in the risk of experiencing poverty and exclusion.
- Health status indicators have long been accepted as major tools to measure social progress over time and countries, e.g. through the Human Development Index and the Human Poverty Index among many others. Health outcomes can include indices of mortality, morbidity, and ability to function. The most widely used health outcome is longevity at birth (which allows reliable comparability across countries and years), as ‘one major indicator of human poverty is a short life’ (Sen & Anand 1997). Life expectancy is therefore adopted to capture the health dimension of Social Inclusion. Such indicator is not free from limitations, as illustrated by Atkinson et al. (2004). For example, an alternative and perhaps more informative measure would require comparative data on life expectancy in good health (free from disability).

**Table 1.** Variables’ definitions

Variable	Definition
Poverty rate	Share of individuals living in households with an income below 60% national median equivalised disposable income.
Long-term unemployment rate	Total long-term unemployed population (≥12 months; ILO definition) as proportion of total active population.
Early school-leavers	Share of total population of 18–24-year-olds having achieved ISCED level 2 or less and not attending education or training.
Life expectancy at birth	Number of years a newborn person may be expected to live.

We choose administrative regions as the main territorial unit of this analysis, with the aim of capturing higher variability than it can be inferred from aggregate national data. Data availability is a serious constraint for analyses which focus on administrative regions, in a wide set of countries and for a long time-period (Lefebvre et al., 2010; Chiappero-Martinetti & von Jacobi 2012). In particular, the Social Inclusion variables are available in the Eurostat Regional Database for 63 administrative regions in five countries (Belgium, Denmark, Germany, Italy, and Spain), for years 2004–2017 (data for Denmark start from year 2006).^2^

As summarised in Table 2, Italy and Spain exhibit the highest overall longevity levels, but they fare substantially worse in the economic and educational dimensions, besides showing a larger regional heterogeneity. School dropouts are relatively low in Denmark Belgium and Germany. However, the worst performing regions in Belgium and Germany (Bruxelles and Saarland, respectively) exhibit dropouts larger than 20%. In Italy and in Spain, the range between best and worst regions amounts to 26 percentage points (Italy), and 45 percentage points (Spain). Similarly, although average long-term unemployment is similar across the five countries (with the lowest levels recorded in Denmark), regional heterogeneity is substantially higher in Italy and Spain. Average poverty rates are higher in Italy and especially in Spain, where again regional divides are substantial.

**Table 2.** Descriptive statistics for four indicators of social inclusion, years 2004–2017

		Belgium	Denmark	Germany	Italy	Spain
Longevity (in years)	Min	77.5 Wallonie ‘04	77.6 Sjælland ‘07	77.8 Sachsen-A. 04	79.5 Campania ‘05	78.3 Ceuta ‘04
	Average	79.8	79.2	80.2	82	81.4
	Max	82.5 Vlaanderen ‘17	81.5 Midtjylland ‘16	82.2 Baden-W. ‘14	84.4 TAA ‘17	85.2 Madrid ‘16
Early school leaving (in %)	Min	6.8 Vlaanderen ‘16	5.7 Hovedstaden ‘16	5.4 Thüringen ‘09	6.7 Umbria ‘16	7 P. Vasco ‘16
	Average	12.1	11.1	12.3	18.4	29.3
	Max	20 Brussels ‘07	16.7 Sjælland ‘09	21 Saarland ‘05	32.7 Sardegna ‘05	54.2 Ceuta ‘05
Long-term unemployment (in %)	Min	1.4 Vlaanderen ‘08	0.3 Midtjylland ‘08	0.7 Bayern ‘17	0.5 TAA ‘04	0.7 Navarra ‘06
	Average	3.8	1.2	4.1	3.6	4.8
	Max	11 Brussels ‘15	2.4 Syddanmark ‘12	13.8 Sachsen-A. ‘04	15.7 Calabria ‘14	22.9 Ceuta ‘14
At-risk-of-poverty rate (in %)	Min	9.8 Vlaanderen ‘11	7.8 Sjælland ‘15	10 Baden-W. ‘07	5.2 VDA ‘06	5.3 Navarra ‘07
	Average	14.8	12.6	14.5	16.9	20.8
	Max	33.7 Brussels ‘11	16.8 Nordjylland ‘13	24.8 Bremen ‘15	44.3 Sicilia ‘11	48.9 Ceuta ‘08

Eurostat Regional Database 2005–2017. Country labels correspond the ISO 3166-1 alpha-2 standard.

VDA, Valle d’Aosta (Italy); TAA, Trentino—Alto Adige (Italy).

Figure 11 (in online supplementary material, Appendix 1.1) summarises the time trends for the four indicators, highlighting converging (and improving) trends for school dropout rates, parallel (improving) trends for longevity, and diverging trends for poverty- and unemployment-rates. Table 3 includes the correlation coefficients among the four indicators of Social Inclusion.

**Table 3.** Correlation matrix for the four indicators of social inclusion

	Longevity	School leavers	LT unemployment	Poverty rate
Longevity	1			
School leavers	−0.025	1		
LT unemployment	0.112	0.276	1	
Poverty rate	−0.142	0.497	0.643	1

## 3. Methods

### 3.1 The Choquet integral as aggregation operator

Hereafter, we assume that Social Inclusion can be described with a bounded cardinal indicator, generated by a function *W*, having a vector of attributes as its arguments. The function *W* needs to be expressive enough to approximate its target sufficiently well, and not overly flexible, to avoid poor generalisation performance (Hüllermeier & Schmitt 2014). While *W* has been often characterised as a linear function (e.g. Peiró-Palomino (2018)), which is convenient for both implementation and dissemination purposes, there is a growing consensus that a linear model lacks sufficient expressiveness, as it imposes a priori perfect compensation and no-interaction among attributes, through the ‘preference independence assumption’ (the total is equal to the sum of the parts). This assumption is theoretically at-odds with the concept of Social Inclusion, where attributes should be characterised by positive or negative interactions: for example, a set of attributes with homogeneous performances might be socially preferred to a set with some attributes scoring very high and some very bad (Angilella et al., 2014; Klugman, Rodríguez & Choi 2011; Krishnakumar 2018, Meyer & Ponthière 2011). Thus, scholars have proposed non-compensative monetary (e.g. Daly et al., (1994); Decancq & Schokkaert (2016)) and non-monetary aggregation functions, e.g. through the CES framework (Bourguignon & Chakravarty 2003; Krishnakumar 2018) in its *geometric* (Klugman et al., 2011; Rogge & Konttinen 2018), *minimum-operator* form (Krishnakumar 2018), and further flexible alternatives (Mazziotta & Pareto 2016), or through the counting approaches (Alkire & Foster 2011). However, these frameworks do not allow to identify an interaction-coefficient for any n-tuple of attributes, nor they can assign weights to coalitions of attributes (Meyer & Ponthière 2011). For example, the CES framework allows to allocate weights to each attribute separately, and to define a one-fit-all constant measure of ‘tolerated’ substitutability, without a direct control of the interaction among any n-tuples of attributes.^3^

To overcome this limitation, we employ Non-Additive Measures (NAM) and the Choquet Integral as aggregation operator (see also Grabisch et al., (2008); Grabisch & Labreuche (2010) and Meyer & Ponthière (2011) for further details). Given that a measure is assigned to each n-tuple of attributes, Choquet integral allows to represent many preferences structures, ranging from the arithmetic/weighted mean to the minimum and maximum operator. Yet, although the Choquet Integral has been used in the economic literature on inequality (Gajdos 2002), environmental sustainability (Pinar et al., 2014), well-being (Bertin et al., 2018; Meyer & Ponthière 2011), and customer satisfaction evaluation (Angilella et al., 2014), it has never been applied, to the best of our knowledge, to multidimensional poverty measurement.

We now summarise some fundamental properties of the Choquet integral and of fuzzy measures. A fuzzy measure (also *capacity*), defined over the index set of criteria N={1,2,…,n}, is a set function $\mu \;:\;2^{N}\to [0,1]$ satisfying the following boundary and monotonicity conditions:


(1) $$\{\begin{array}{c} \mu \{\emptyset \}=0\\ \mu \{N\}=1\\ \mu \{S\}\leq \mu \{T\}\leq 1,\;\;\forall \;S\subseteq T\subseteq N\; \end{array}$$

Such non-additive measure (NAM) assigns to every subset (coalition) of criteria a measure which can be greater, smaller, or equal than the sum of the measures of their singletons, depending on whether the criteria in the coalition are characterised by synergic-, redundant-, or no-interaction. In the latter case, the NAM collapses to the linear weighted aggregation (WA). Given a NAM *μ*, its Möbius representation is a set function such as the following (Marichal 2000):


(2) $$m\{S\}=\underset{T\subseteq S}{\sum }\;(-1{)}^{s-t}\mu \{T\},\;\;\forall S\subseteq N,\;s\leq k$$

where s=card{(S)}, t=card{T}.

The inverse of 2) is called *zeta transformation* and is given by:


(3) $$\mu \{S\}=\underset{T\subseteq S}{\sum }\;m\{T\},\;\;\forall S\subseteq N,\;t\leq k$$

The boundary and the monotonicity conditions are given by the following constraints (Marichal 2000):


(4) $$\{\begin{array}{c} m\{\emptyset \}=0\;\\ \underset{T\subseteq N}{\sum }\;m\{T\}=1\\ \underset{\begin{array}{c} T\subseteq S\\ T∋i \end{array}}{\sum }\\ m\{T\}\geq 0,\forall S\subseteq N,\;\;\;\forall i\in S \end{array}\;$$

Let *μ* be a NAM defined on *N* and $X=\{x_{1},x_{2},\ldots ,x_{n}\}$ the normalised values of the criteria belonging to *N*; the (discrete) Choquet integral with respect to *μ* is defined as follows (Grabisch 1997):


(5) $$C_{\mu }(x_{1},\ldots ,x_{n})=\sum_{i=1}^{n}(x_{\left(i\right)}-x_{\left(i-1\right)})\mu \{A_{\left(i\right)}\}$$

where (i) means that the indices have been permutated in such a way that $x_{\left(1\right)}\leq \ldots \leq x_{\left(n\right)}$, while $A_{\left(i\right)}=\{x_{\left(i\right)},\ldots ,x_{\left(n\right)}\}$ and $x_{\left(0\right)}=0$. Using the Möbius representation the Choquet integral can be written as:


(6) $$C_{m}(x_{1},\ldots ,x_{n})=\sum_{T\subseteq N}m\{T\}\wedge_{i\in T}x_{i}$$

being ∧ the minimum operator.

To define a capacity *μ* on *N*, $2^{N-1}$ parameters are required.^4^ For this reason, such operator is extremely flexible and allows to represent and model, ex ante, many preferences’ structure with respect to the analysed criteria, ranging from perfect complementarity (minimum operator) to perfectly substitutability (maximum operator), passing through the preference independence of the criteria (the arithmetic mean and its weighted version).

Such capacities need to be directly set by one or more Decision-Makers or implicitly elicited by means of a suitable questionnaire. The ex ante model-ability is extremely important, because—in the panel experts’ preference elicitation stage—it does not impose a priori any particular functional form that may be in contrast with the interviewee’s opinions.

#### 3.1.1 Shapley values, interaction index, Orness/Andness index

In order to enhance the interpretation, summarisation and description of fuzzy measures, we will exploit three widely used behavioural indices: the *Shapley* value (Shapley 1953), the *Interaction* index and the *orness index* (or its opposite version *andness index*) (Grabisch 1997; Murofushi & Soneda 1993).

The Shapley value is a measure [0,1] of the relative importance of an attribute (criterion), considering all the marginal gains in Social Inclusion between any coalition not including the attribute, and those which include it. In terms of Möbius representation, the Shapley value of criterion *i* is defined as following:


(7) $$\varphi_{m}(i)=\underset{T∋i\;}{\sum }\frac{1}{t}m\{T\},\;\;T\subseteq N$$

with t=card(T).

The Interaction index [−1,1] among a combination S⊆N of criteria with card(S)≥2, represents the degree of complementarity (1) or substitutability (−1) in the coalition; when two criteria are independent, the interaction index equals zero. Its Möbius representation reads as follows (Grabisch 1997):


(8) $$I_{m}(S)=\underset{T⊇S}{\sum }\frac{1}{t-s+1}m\{T\},\;\;T\subseteq N$$

The Orness/Andness degree [0,1], similarly to the interaction index, represents the degree of whole substitutability between the *n* criteria; it is a measure of the tolerance of the decision–maker’s preferences with respect to the criteria proposed. Indeed, tolerant decision-makers (Orness>0.5) can accept that only some criteria are satisfied. The higher the tolerance, the closer the aggregation function is to the ‘maximum’ operator. On the other hand, intolerant decision-makers demand that most criteria are satisfied. This corresponds to a conjunctive behaviour (Orness<0.5), whose extreme case is the ‘minimum’ operator. When the *n* criteria are additive, we have that Orness=Andness=0.5.

By construction:


(9) Orness=1−Andness

(10) $$Orness=\frac{1}{n-1}\underset{T\subseteq N}{\sum }\frac{n-t}{t+1}m\{T\}$$

with n=card(N).

### 3.2 Expert-based approach and sample selection

The strategies to determine the parameters of an aggregation function are commonly divided between ‘positive’ and ‘normative’ (see Decancq & Lugo (2013) for a discussion and literature review). Positive (or ‘descriptive’) methods include data-driven approaches such as Principal Component Analysis (PCA) and Data Envelopment Analysis (also known as Benefit of the Doubt, BoD). Such statistical methods are often adopted because they do not require the availability of ‘objective knowledge on the true policy weights’ (Rogge & Konttinen 2018). Yet, their independence from policy or economic judgement limits the possibility to interpret their results from a normative perspective (an impossibility often referred to as the ‘Hume’s guillotine’, see Decancq & Lugo (2013)). The BoD method assigns weights in order to maximise an underlying optimisation function. An implication of this method is that several criteria can be assigned zero, or very low, weight (as in the work by Rogge & Konttinen (2018) on Social Inclusion). The PCA method (e.g. (Döpke et al., 2017; Ivaldi et al., 2016), which assigns weights on the basis of the observed correlation between attributes, has also been described as less suitable for policy evaluation, due its weights having statistical, yet not economic, justification (Decancq & Lugo 2013; Mazziotta & Pareto 2019).

Normative methods aim to characterise the aggregation parameters with explicit and economically meaningful preferences, either set by the researcher (e.g. equal weighting), or by some policy targets, or derived from participatory methods (e.g. experts’ opinions). In the context of Social Inclusion, there are no comparable policy goals adopted at the European level, as countries typically set national poverty and social exclusion targets (Social Protection Committee 2018). In the latter case, the choice of the expert sample is arbitrary, and it is often subject to trade-offs between resource-availability, degree of panel-expertise, and representativeness of panel, which can affect the interpretation of the results (Kim et al., 2015).

In this study, we adopt a normative, expert-based, strategy to elicit the Choquet aggregation parameters. As many other authors in the field of multidimensional measurement, we build our work against the background of a well-accepted belief perhaps most famously expressed by Sen & Anand (1997): there are inescapable elements of subjectivity (judgement) in any stage of creating a multidimensional index, from the choice of attributes to dimensions, to aggregation weights, to data normalisation. Therefore, a crucial feature of any index, Sen and Anand argue, relies on whether such subjectivity is explicitly stated, so that public scrutiny can occur. Following this rationale, we acknowledge that any weighting scheme we elicit from an experts panel is subjective, for example, because the choice of experts is arbitrary: just as for the parameters in data-driven approaches, the preferences expressed by the expert sample cannot be considered as ‘objective’, and it is possible that different expert panels would lead to different elicited weights.

We therefore adopt four steps in order to generate a composite index characterised by normative-interpretable results and transparent weights, while minimising the subjective bias.

First, we have selected a sample of experts from a homogeneous network of policy makers holding homologous positions and expertise in the field of social inclusion experts.

We chose as experts the 20 Directors General for Social Policies (DGSP) in Italy (*Direttore per le Politiche Sociali/Programmazione sociale*). The DGSPs hold an administrative (i.e. not elected) top managerial role at regional level in Italy, and are responsible for planning and coordinating social policies at local level, with a particular focus on the interchanges between poverty reduction, labour market inclusion and health-care policies, i.e. the core policy-areas of Social Inclusion.^5^ The choice of a specific and homogeneous expert-sample should ensure a high level of ex ante internal consistency, as the experts are: (i) public managers at regional level in Italy; and (ii) they all oversee the planning and coordination of social policies. This should reassure the reader that, although individual competences can vary greatly, our experts all share a broad perspective and expertise on Social Inclusion, hence allowing us to collect relevant, informed, and comparable policy-preferences on Social Inclusion. Moreover, being the population of DGSPs limited, representativeness is easier to achieve.^6^

Several alternatives for preference elicitation existed, several of them would require additional resources, including surveying a random population (Kristensen & Johansson 2008; Pouliakas & Theodossiou 2010), students (Meyer & Ponthière 2011), or exploiting secondary data from large surveys (Decancq et al., 2019; Decancq & Schokkaert 2015; Noble et al., 2008). These strategies were unfeasible, as they would not have allowed us to collect preferences from participants with comparable and extensive experience in Social Inclusion. Alternatively, we could have selected a heterogeneous set of experts, i.e. prominent experts in different areas of the public or private sector (Bertin, Carrino & Giove 2018; Chowdhury & Squire 2006; Cohen & Saisana 2014; Hoskins & Mascherini 2009; Pinar et al., 2014). However, this would reduce out ability to reach a good representativeness of the chosen expert population.

Second, as recommended by the relevant literature (e.g. Kristensen & Johansson (2008); Decancq & Lugo (2013)) we normalised the core statistical variables which are then used in the preference elicitation exercise. There are two reasons for this choice, as discussed in section 3.3.3. First, to fulfil the ‘scenario equivalence’ assumption, which states that the attribute levels in any scenario should be understood in the same way by all experts. Second, because our aim is to build a normative measure of Social Inclusion, the unit of measurement of its normalised attributes must reflect some value judgement (an expert-based normalisation function) (Carrino 2016; Chowdhury & Squire 2006; Despic & Simonovic 2000; Hoskins & Mascherini 2009; Meyer & Ponthière 2011; Pouliakas & Theodossiou 2010). As detailed further in online supplementary material, Appendix 1.2, in this paper we employ a min-max normalisation function estimated by Carrino (2016, 2017) through interviews of 149 academics and researchers from the Department of Economics and Management at the Ca’ Foscari University of Venezia (Italy).

Third, we present and discuss the extent to which our experts’ elicited weights reflect a consensus (Section 4.1). While our experts will be shown to have similar preferences in terms of aggregation weights, we prudently recall that no ‘true values’ of the weights exist. In the words of Mascherini & Hoskins (2008), ‘the judgment of one of the outliers may be correct, and those who share a consensus view may be wrong’.

Fourth, as sensitivity analysis (Section 4.3), we show the distribution of our Social Inclusion index across all the experts (that is, we repeatedly compute Social Inclusion for each European region by adopting the weights-set of one expert at a time, and then we show the distribution of the obtained values for Social Inclusion).

### 3.3 Preferences’ elicitation approach and survey design

#### 3.3.1 Preferences’ elicitation approach

In order to estimate the parameters (capacities) of the Choquet Integral, we follow the Least-Squares capacity-elicitation approach, a widely used c*ardinal*-based fuzzy measure elicitation method which allows to identify the values of the Möbius coefficients (and thus the behavioural indices as *Shapley*, *interaction*, *orness*) from the answer given by one or more experts to a suitable designed questionnaire (see Section 3.3.2).^7^ For example, an expert is submitted a questionnaire formed by *v* questions. Each question represents a *j*th hypothetical scenario, constituted by a vector $\left[x_{1}(j),\ldots ,x_{n}(j)\right]$ of criteria values (normalised between 0 and 10). The expert then provides an evaluation y(j) (in the same scale). The least-squares method aims at minimising the average quadratic distance between the expert’s evaluations provided and the predicted values computed by means of the Choquet integral.

The Least-Squares problem has a unique solution—i.e. the quadratic program is strictly convex—if and only if the criteria values attached to the scenarios are properly chosen (for details, see Farnia & Giove (2015)). Bertin et al. (2018) (p.26) provide a detailed example of how the Least-Squares estimation is performed in a setting similar to ours.

#### 3.3.2 Survey design

The scenario-evaluation is a well-established method to retrieve a respondent’s preferences on the known attributes of a complex phenomenon, based on the answers she gives to a specific questionnaire (Benjamin et al., 2014; Bertin et al., 2018; Despic & Simonovic 2000; Green & Rao 1971; Kristensen & Johansson 2008; Meyer & Ponthière 2011; Pinar et al., 2014; Scholl et al., 2005). This method, an application of conjoint analysis, is preferred to the widely used strategy of budget-allocation (Chowdhury & Squire 2006; Hoskins & Mascherini 2009; Kim, Kee & Lee 2015) which, by assigning weights to each attribute independently, imposes an a priori assumption of no interaction.

Through a face-to-face questionnaire, we presented each decision-maker with a finite number of scenarios depicting hypothetical societies (vignettes). Each scenario is described by a vector involving different levels of the four core attributes for employment, health, income, and education. Respondents evaluate each scenario on a stepwise cardinal scale from 0 to 10. The evaluation requires to consider all the attributes at once, and to trade them off in order to produce an overall evaluation. Thus, respondents implicitly implement their personal ‘welfare function’, as they would when facing a real scenario.

We assumed that each attribute in a scenario (specifically: longevity, long-term unemployment, poverty rate, school dropouts) can take three performance-levels, i.e. High (corresponding to a score of 10), Intermediate (5) or Low (0). We then built a full-rank matrix of 27 scenarios, which ensures the unicity of the solution to the estimation of the Choquet parameters (see Section 3.3.1).^8^

For this exercise to be valid, we needed to satisfy the crucial ‘scenario equivalence’ assumption (Kristensen & Johansson 2008), stating that the attribute levels in each scenario should be understood in the same way by all respondents. We took several steps to make this assumption believable.

First, as suggested by the literature on expert elicitation, we provided participants with examples of what was desired in clear and simple language, to guide them through the process (Cohen & Saisana 2014). Second, we implemented five trivial scenarios (where all attributes, and the overall outcome, were set at either 0 (low), 2.5 (mid-low), 5 (intermediate), 7.5 (mid-high), or 10 (high)), to enhance answers’ consistency: respondents were asked to drag each scenario near the trivial scenario which they thought was most representing the embedded level of Inclusion.^9^ Third, as discussed in the previous Section, we drew respondents from a homogeneous network of policy makers holding homologous positions and expertise in the field of social inclusion.

**Figure 1.**
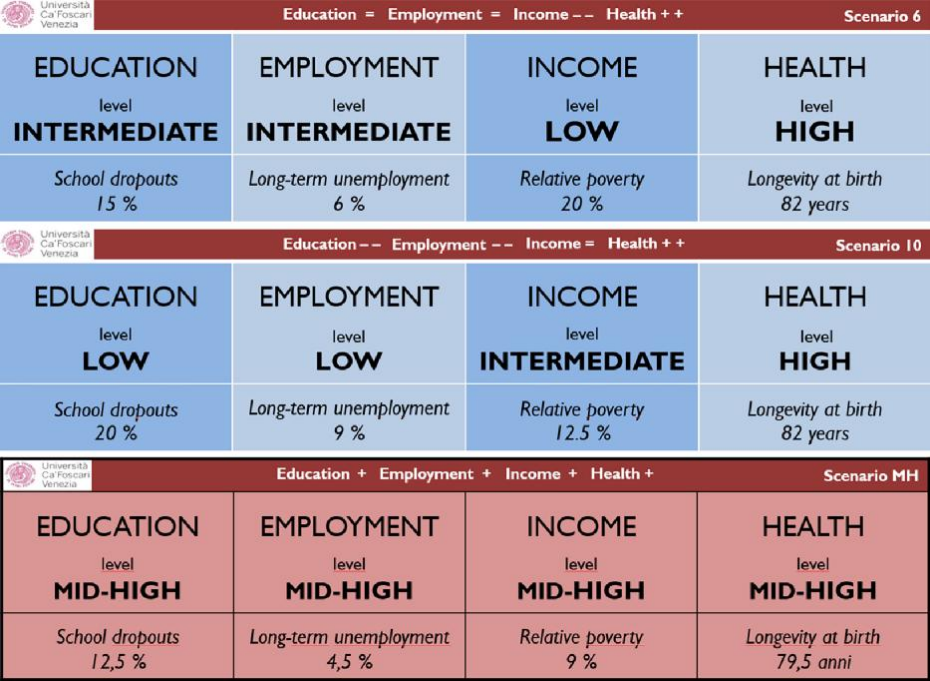
Examples of scenarios.

Fourth and foremost, the decision matrix was built so that each attribute-level was characterised with a specific numerical example, following a normalisation procedure described hereafter. Figure 1 illustrates some of the cards used to depict scenarios (here translated in English).

A limitation of this approach relies on the limited number of attributes that a decision-maker can simultaneously deal with in order to elicit consistent answers. This could become an issue for studies employing numerous attributes (e.g. Peiró-Palomino (2018)). However, a possible solution would be to split the attributes set in conceptually meaningful groups, as done by, e.g. Bertin et al. (2018).

#### 3.3.3 Normalisation strategy

To avoid that each respondent interprets the verbal label differently, we characterised the labels with a specific numeric example (Despic & Simonovic 2000; Pouliakas & Theodossiou 2010). This required us to normalise each attribute from its original scale to a fixed 0–10 scale where 0 and 10 correspond to, respectively, a ‘very bad’ and an ‘excellent’ performance, and 5 is ‘intermediate’ (Meyer & Ponthière 2011). We employ the linear min–max normalisation function (Giovannini et al., 2008), which rescales variables between 0 and 10 depending on how far they are from a low and a high threshold. Adopting a data-driven approach in setting the thresholds (e.g. choosing the observed minimum and maximum performance as thresholds, as in Peiró-Palomino (2018)) leads to a unit of measurement which is free from a normative connotation, yet it is harder to determine what does it reflect in economic terms (Carrino 2016; Lefebvre et al., 2010). If the parameters are data-driven, then the normalised variables are suitable to be interpreted under a statistical perspective. As an example, in the data-driven min–max, a variable with transformed-value equal to ‘0’ just implies it being ‘the last one’, or ‘the worst one’ observed among the available data, which does not necessarily correspond to an undesirable condition of poverty. As our aim is to build a normative measure of Social Inclusion, the unit of measurement of its normalised attributes must be normalised according to some value judgement (an expert-based normalisation function). This translates to linking the extreme values ‘0’ and the ‘100’ with, e.g. a certain definition of desirability, thus making the normalisation independent from the data. When an indicator lies above or below such fixed ‘goalposts’, further variations do not contribute to the composite measure (see e.g. the discussion in Anand & Sen (1994); Klugman, Rodríguez & Choi (2011); Ravallion (2012); Lefebvre et al., (2010) and Mazziotta & Pareto (2016)).

In this paper, we follow the recommendations from the United Nations Development Programme and adopt ‘desirable’ and ‘undesirable’ targets as benchmarks, which allows us to characterise the normalisation function in a normative way. Specifically, we exploit the thresholds elicited by Carrino (2016, 2017) from a large homogeneous sample of Economics scholars at the Ca’Foscari University of Venezia (Italy). The normalisation strategy is detailed in online supplementary material, Appendix 1.2.

### 3.4 Experts’ preferences aggregation

Our methodology would, in principle, allow us to estimate a Choquet Integral for each decision maker, which would result in as many indices of Social Inclusion. However, to ease the interpretation of the output, we build a ‘representative’ expert (Expert Fusion), and its related Inclusion index. Many approaches can be used for the fusion of Experts’ preferences, and many of them are based on a consensus procedure (a non-exhaustive reference can be Herrera-Viedma et al., (2002); Li et al., (2014); Quesada et al., (2015)). In this context, we apply an approach similar to Farnia & Giove (2015), and we weight the experts’ answers based on their consistency in answering the questionnaire (rather than, e.g. on how much their answers are close to the average answer of the other decision-makers). In our perspective, the fact that an expert has a strong dissenting opinion compares to the remaining sample is not, per se, a reason to reduce its contribution to the Fusion expert. It is, however, important to detect potential cases of inconsistency, randomness, etc., in the evaluation process.

Given that NAM approach is sufficiently general to cover many preference structures, Expert’s preference has been weighted according to his/her overall consistency in judging the alternatives proposed in the Choquet context. We measure Expert’s consistency as a function of the sum of squared distances, in such a way that the greater (smaller) the sum, the smaller (greater) the contribution from the relative Expert.

Given *v* alternatives to be judged, we compute the $R^{2}$ index for each *j*th Expert:


(12) $$R_{j}^{2}=\frac{\sum_{i=1}^{v}\;{\left({\hat{y}}_{\;ji}-{\bar{y}}_{j}\right)}^{2}}{\sum_{i=1}^{v}\;{\left(y_{\;ji}-{\bar{y}}_{j}\right)}^{2}}$$

where ${\hat{y}}_{\;ji}$ represents the estimated Choquet value for expert *j*th in the *i*th scenario, $y_{\;ji}$ the Choquet value set by the expert *j*th in the *i*th scenario and ${\bar{y}}_{j}$ the sample mean of all his/her decisions.

The weight attached to the preference of the *j*th Expert is computed as follows:


(13) $$w_{j}=\frac{R_{j}^{2}}{\sum_{\;j=1}^{d}\;R_{j}^{2}}$$

Given that a linear weighted combination of Möbius representations is a Möbius representation too, the final Möbius representation for the Expert Fusion can be defined in the following:


(14) $$m^{*}\{T\}=\overset{d}{\underset{\;j=1}{\sum }}\;w_{j}m_{j}\{T\}\;\;\forall T\subseteq N$$

While we adopt the aforementioned method in the main analysis, we have verified that the results of our Social Inclusion index are almost identical when we use an alternative aggregation strategy of the experts’ preferences, i.e. a simple average (results available in the online supplementary material, Appendix 1.2).

### 3.5 On measurement error

In principle, both the conceptual framework and the empirical framework for Social Inclusion are vulnerable to measurement error. Indeed, the conceptual framework of Social Inclusion was introduced exactly because of the measurement error that was inherent to the theoretical approaches that essentially equated Social Exclusion to poverty. As explained by Atkinson et al., (2004), measurement error is one of the raison d’être of Social Inclusion: as income data are inadequate to fully capture social disadvantages, non-monetary dimensions have been included to supplement information on income. As discussed in Section 2, however, the current EU’s theoretical framework on Social Inclusion is not free from limitations, and alternative frameworks have been proposed. Nevertheless, we believe that a large number of contributions in economics, public policy and sociology since the work of the Atkinson Commission have shown that the EU’s definition of Social Inclusion is a solid, and theoretically grounded, step in the right direction towards measuring social disadvantage.

From an empirical perspective, we emphasise that the statistical indicators are not immune from measurement error (although their choice was also guided by the aim of reducing the risk for measurement error to begin with). For example, poverty rates assume that financial resources are equally divided among all those living in a household, ignoring further sources of inequality such as sex-based inequalities, in that women might be largely disadvantaged up to being at risk of poverty than men, even though the household as a whole is not (Atkinson et al., 2004). Indicators on housing have been excluded from the original empirical definition of Social Inclusion, due to a lack of data quality, such as data unavailability and absence of a common set of definitions and measures, especially on homelessness and precarious housing (Atkinson et al., 2002; Atkinson et al., 2004). Further limitations can be discussed for health, education, and labour market indicators.

Still, we believe the bias induced by measurement error on the interpretation of our empirical results is limited, for three main reasons. First, we interpret our index in terms of levels of performance rather than in terms of regions ranking. This is because of an explicit principle stated by the Atkinson commission with respect to how the indicators should be used, to limit the bias of measurement error, among other reasons. The authors note that rankings between countries are more vulnerable to measurement errors, while levels of performances are more robust (Atkinson et al., 2004). Consistently with this caveat, we will restrain from emphasising small differences across countries in our Social Inclusion index.

Second, if we assume that data measurement error improves or worsens simultaneously for all countries (a plausible hypothesis given the ex ante effort made in the choice of comparable indicators), we can more confidently focus on trends within countries, and on substantial widening or narrowing gaps across countries, such as the gap emerging between Mediterranean regions (on average) and Northern regions (on average).

Third, we note that, similarly to previous works in this literature, our paper is mostly focused on the aggregation methodology, meaning the interpretation of normalisation and aggregation strategies. Our aggregation method (as our normalisation method) is relatively less subject to measurement error than data-driven methods, as the weights are derived from experts’ preferences based on fictional scenarios, where there is no measurement error. Data-driven parameters (e.g. weights) are derived from the observed performances, therefore being potentially more exposed to measurement error bias than expert-driven parameters.

Nevertheless, the measurement errors in the statistical attributes of the Social Inclusion index constitute a relevant issue when discussing the results of any index, and further research is surely needed to clarify and minimise the related induced bias.

## 4. Results

### 4.1 Decision-makers’ preferences

Twelve Experts were interviewed to gather preferences on the criteria considered in the composite index. In this section, the results of the decision process are shown, with a focus on the main behavioural indices listed in section 3.1; full results are available in online supplementary material, Appendix 1.4, Table 6.

**Figure 2.**
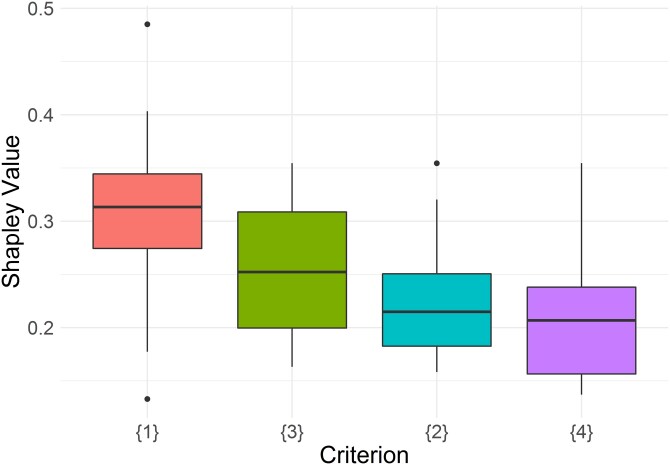
Estimated DM shapley values for the four attributes of social inclusion. (1), Education; (2), L.T. Unemployment; (3), Poverty; (4), Life expectancy.

Referring to Shapley values, hence to the relative importance of each criterion, Figure 2 shows that Experts consider *education* as the main driver of social inclusion (preference’s fusion value 0.306), followed by *poverty* (0.257), *unemployment* (0.226), and *life expectancy* (0.209). Interestingly, education represents the most important criterion for the ‘representative expert’, while also exhibiting the lowest degree of consensus among Experts, measured in terms of volatility of preferences. Although outliers, the minimum and maximum values are 0.484 and 0.132, respectively. Poverty, unemployment, and life expectancy share a similar level of consensus in terms of volatility. In the main analysis, we will aggregate experts’ preferences in a Fusion expert preference set. However, in sensitivity analysis, we will build a specific index of Social Inclusion for each expert. We will do so because, as discussed earlier in the text, while we can value consensus as a hint to the robustness of preferences ‘the judgment of one of the outlier may be correct, and those who share a consensus view may be wrong’ (Mascherini & Hoskins 2008), and we would need a more sophisticated participatory method (such as a Nominal Group Technique as in Bertin et al., (2018)) to disentangle the extent to which experts’ opinions are really diverging.

**Figure 3.**
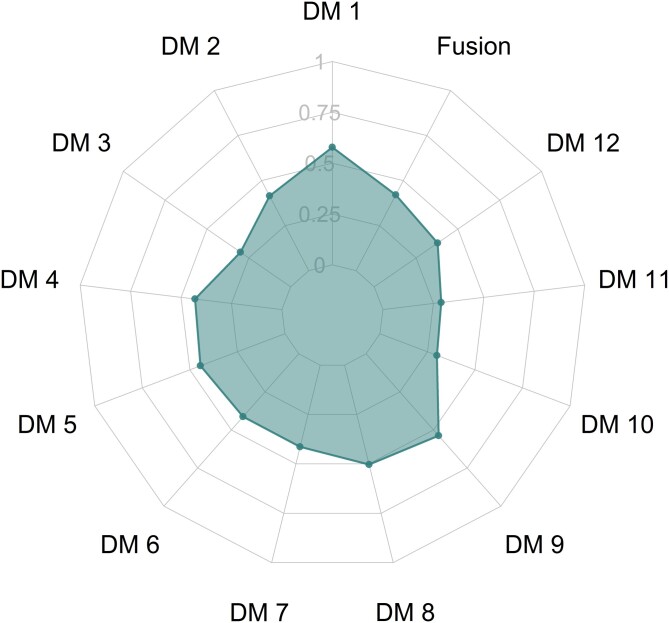
Orness index from the DM elicited preferences.

There is no strong overall evidence of strong complementarity or substitutability among criteria, as represented by the *orness* index (value 0.42; see Figure 3). The results show that in the case of Experts preferences’ fusion there is indeed only a slight preference for complementarity. As the *orness* index varies in the range [0,1] with 0.5 indicating preferential independence among criteria, Experts’ preferences vary from remarkable complementarity (0.288) to light substitutability (0.578).

**Figure 4.**
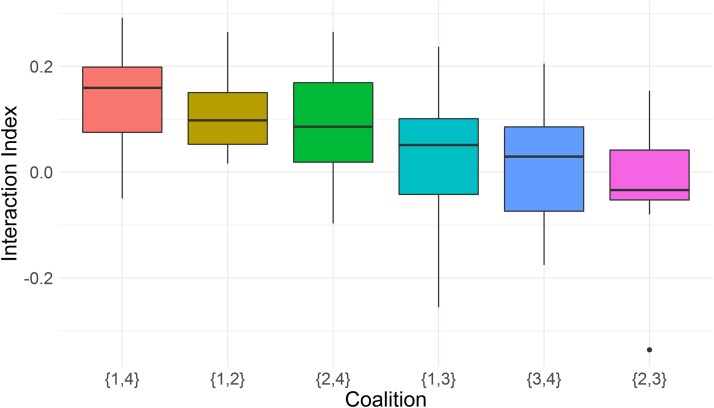
Distribution of interaction coefficients from DM elicited preferences. (1), Education; (2), L.T. Unemployment; (3), Poverty; (4), Life expectancy.

Interaction indices (Figure 4) allow to evaluate more in details the degree of complementarity or substitutability among couples of criteria. Such index varies in the range [−1,+1] where −1 represents perfect substitutability and +1 perfect complementarity.

Education & life expectancy, education & unemployment, unemployment & life expectancy represent the three couples of criteria with the highest level, although not remarkable, of complementarity; while in the first and third case, all Experts but one consider them slightly complementary criteria, in the case of education & unemployment all experts agree to not consider them substitutes. The latter is the case where Experts have shown the largest consensus among them. In the other combinations of criteria, we highlight a general low consensus among Experts given that their preferences swing around the independence case; some of them have considered such criteria more complementary than substitutes, others have considered them in the opposite manner. This clearly can be seen in the case of education & poverty or in the case of unemployment & poverty, where preferences swing around zero.

### 4.2 The social inclusion index

#### 4.2.1 Social inclusion Index by country

**Figure 5.**
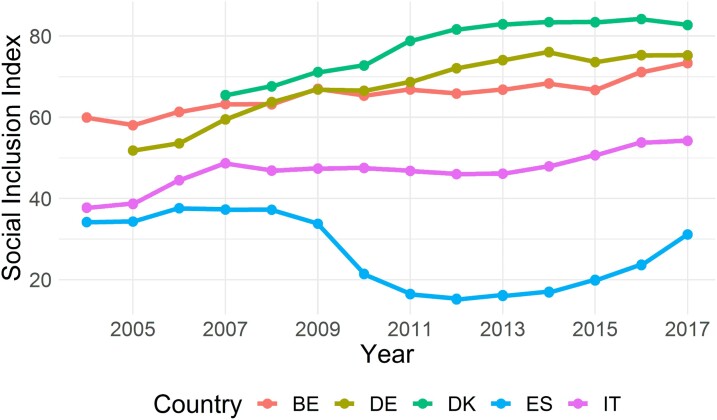
Social inclusion Index with preferences from the ‘Fusion DM’, country averages (population weighted). The figure plots the average Social Inclusion score by country and year, based on the ‘Fusion DM’ preferences.

We start by showing the Social Inclusion Index computed according to the preferences of the ‘Fusion’ Expert (details in Section 3.4). We aggregate regional Social Inclusion scores into country figures (regional scores are weighted by population size within a country) and summarise them in Figure 5. Because of the normalisation strategy we employed, the levels of Social Inclusion can be fully interpreted as carrying value judgements, in that low levels of Inclusion convey an undesirable social condition, while high levels convey a desirable social condition. Finally, due to the potential underlying measurement error in the original statistical indicators which the index bases upon, we restrain from commenting upon small differences in levels of performance across countries.

Our results can be summarised in three main parts. First, the lowest levels of Social Inclusion are found in Italy and Spain, at any time, while Denmark exhibits the highest scores since 2010. There is no point in time during the considered interval where Italy or Spain reached the same level of Social Inclusion of Belgium, Denmark or Germany.

Second, the gap in Social Inclusion among the five countries, in particular between the Mediterranean countries and the Continental countries, increases dramatically during the observed time interval. This suggests that a polarisation has taken place during the last 15 years, in that most regions in Spain and Italy have seen their Social Inclusion either declining or stagnating, while other European regions were improving their conditions.

Third, dynamics are rather different across countries. Denmark and Germany exhibit an improvement in Social Inclusion even in the aftermath of the Great Recession. Belgium and Italy, on the other hand, show a flattening curve after the 2008 crisis, with both countries picking up on an increasing trend in 2016 and 2017. Spain’s Social Inclusion drops substantially between 2008 and 2012, although a steep recovery is shown after 2014. However, Spain’s levels of Inclusion in 2017 are far lower than in the pre-2008 years. The trends for Spain and, to a less extent for Italy, show that the Great Recession deeply hurt Mediterranean regions while Continental regions were better able to cope.

All in all, this result conveys a worrisome picture of Social Inclusion in our selected countries. The average evaluation of Social Inclusion in Denmark is more than three times higher than in Spain, in 2017, but this gap reached a factor of four during the previous years.

#### 4.2.2 Heterogeneity in Social Inclusion within country

**Figure 6.**
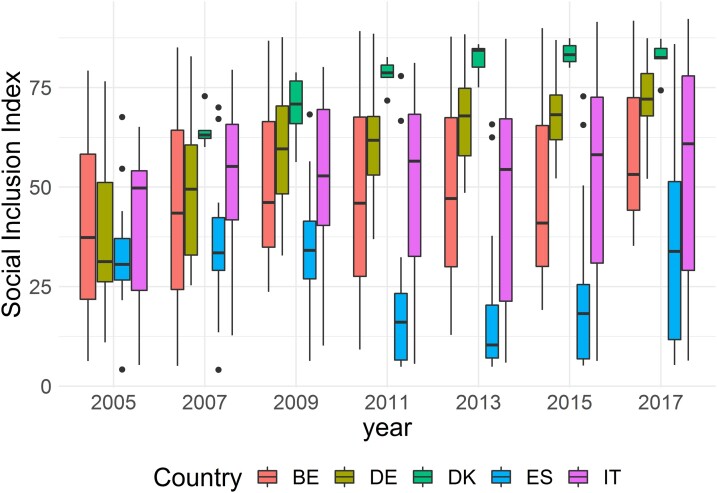
Distribution of regional social inclusion Index within each country (selected years). The median in each box-plot represents the median Social Inclusion value across regions in a country, computed according to the ‘Fusion Decision Maker’ preferences. Such median is not weighted by the regions’ population. Hence, it should not be compared with the average value shown in Figure 5 (which is population-weighted).

By exploiting regional disaggregation, we can elaborate more on the sub-national inequalities in Social Inclusion within countries. In Figure 6, we plot the distribution of regional Social Inclusion by country (for selected years): the larger the boxplot, the larger the regional inequalities. Results indicate that: (i) Denmark’s (high) Social Inclusion levels are consistently homogeneous across its regions; (ii) Germany has seen both an increase in average Social Inclusion, and a reduction of its territorial difference across the time-interval; (iii) the drop in Spain’s Social Inclusion is not accompanied by an increase in its regional variability, which suggests that the decline has been largely widespread across the country, except for the latest year 2017, where the size of the boxplot suggests that the recovery process has been stronger in some areas than in others; (iv) Belgium and Italy exhibit a very high internal variability, which confirms the well-known inequalities between north and south in both countries. Indeed, and especially in the most recent years, the top 25% of Italian regions perform at similar levels than the best-performing regions in Germany, and the same holds for Belgium’s Vlaams. Interestingly, the worst 25% regions in Italy exhibited, after the financial crisis, a Social Inclusion level still higher than the median Social Inclusion levels in Spain, except for in year 2017.

#### 4.2.3 A closer look to regional social inclusion

**Figure 7.**
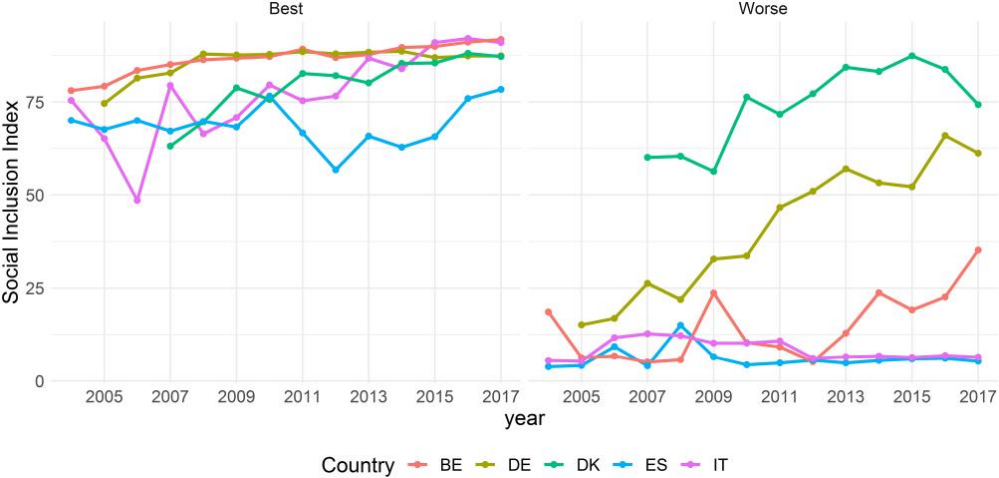
Social inclusion index for best (left panel) and worst (right panel) regions. Best (worst) regions by country. Belgium, Vlaams (Bruxelles); Denmark, Sjaelland (Nordjylland); Germany, Baden-Wurttemberg (Berlin); Italy, Trentino Alto-Adige (Sicilia); Spain, Navarra (Ceuta).

To highlight some dynamics hidden from the previous graphs, in Figure 7, we plot the trends in Social Inclusion for the best (left panel) and worst (right panel) regions in each country (best and worst performance is computed on average across years). Among the best regions, different patterns emerge: (i) while Belgium’s Vlaams and Germany’s Baden-Württemberg exhibit a rather flat trend, Denmark’s Sjaelland sees an almost continuous catching-up in Social Inclusion; (ii) albeit both Italy’s Trentino—Alto Adige and Spain’s Navarra see their Social Inclusion decline after 2008, Trentino shows a quicker convergence with the other best performers than Navarra. A look at the worst performers (right panel) confirms several of the stylised facts already described: (iii) Denmark’s worst region is, for both levels and trends, almost undistinguishable from the ‘best’ regions in the left panel of Figure 7, confirming the high homogeneity of Social Inclusion levels in the country; (ii) Germany’s worst region is steadily improving across the years and clearly departs from Italy’s Spain’s and Belgium’s worst performers in terms of Social Inclusion, which confirms the overall positive trend of Germany observed in Figure 5, and suggests a sort of internal convergence which supports the evidence of declining regional variability shown in Figure 6. Finally, Italy’s and Spain’s worst performers remain steadily on very low and worrisome levels of Social Inclusion, unlike Belgium’s Bruxelles region which shows a clear improvement in its social conditions since 2013, although remaining on levels of Inclusion which are almost three times lower than the worst observed region in Denmark, in 2017.

### 4.3 Sensitivity analysis

#### 4.3.1 Heterogeneity by expert

**Figure 8.**
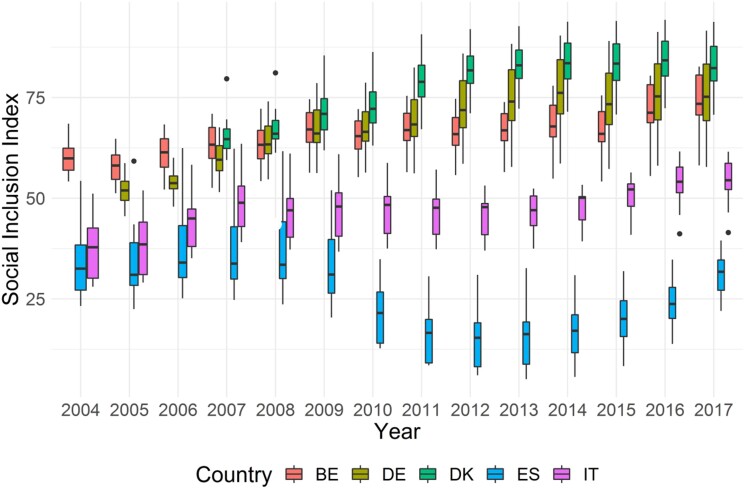
Distribution of the social inclusion indicator across experts. The graph plots the distribution of the Social Inclusion Index by country (regions are aggregated using population weights) across Decision-Makers. The variability within-country is only due to differences in the experts’ preferences.

The index of Inclusion described in the previous section stems from an aggregation function whose parameters are a summary of the preferences of the pool of Experts involved in the elicitation process (‘Fusion expert’). As such, they do not represent the preferences of any specific expert. Thus, one might wonder about the extent to which the Social Inclusion Index changes depending on the preferences of specific experts. We thus re-estimated the Social Inclusion Index separately for each expert and, in Figure 8, we show (for each country and year) the distribution of the values of the Index across all the experts.

Results from this sensitivity analysis confirm the conclusions drawn from the ‘Fusion Expert’ model: the boxplots for Italy and Spain are always lying below those of Germany and Belgium, indicating that, regardless of the experts’ preferences towards the components of Social Inclusion, Mediterranean countries fare worse than continental countries. A similar point can be made for Denmark, whose boxplots always lye above the remaining countries. Belgium and Germany boxplots are overlapping across most of the time interval, indicating that different preferences on the Social Inclusion function might lead to alternatively identify Belgium or Germany as a better performing country.

#### 4.3.2 Partial effects of changes in specific indicators on social inclusion, by country

In our empirical framework, Social Inclusion is a composite index of four specific indicators, described in Section 2. From a policy perspective, it would be valuable to identify which indicators would, if improved, lead to the largest gains on Social Inclusion. We therefore analyse what is the contribution that each indicator brings to the Social Inclusion index, in each country, by estimating how the Social Inclusion index reacts to unitary improvements on normalised indicators of income, education, labour market, and health.

From an analytical perspective, suppose that $\forall T\subseteq N,\;\wedge_{i\in T}x_{i}$ is unique and let $F_{k}=\{T\subseteq N:\wedge_{i\in T}x_{i}=x_{k}\}$ and $v(T)=\wedge_{i\in T}x_{i}$, then the increment in the Choquet integral with respect to criterion $x_{k}$ is given by:


(15) $$\frac{dCh}{dx_{k}}=\underset{T\in F_{k}}{\sum }\;m(T)+\underset{T\notin F_{k}}{\sum }\;m(T)\frac{dv}{dx_{k}}$$

where the first addendum represents the direct effect on the Choquet integral of an increment in the $x_{k}$ criterion; the second term represents its indirect effect. If all criteria are independent from each other, that is $\frac{dx_{j}}{dx_{k}}=0,\;\forall k\neq j$, only a direct effect will exist. In this paper, we prudently restrain ourselves in only computing the direct effect of indicators on Social Inclusion. We believe that, in order to compute the indirect effects, a conceptual model of the causal links between dimensions should be established. Such model has not been detailed so far, to the best of our knowledge, and falls beyond the scope of this work.

We compute the direct effect of a unitary increased in each standardised indicator on the social inclusion index, from the baseline performance measured in 2017 (see online supplementary material, Appendix 1.6, Table 8). We employ the fusion of experts’ preferences to characterise the aggregation function, as in the main analysis.

**Figure 9.**
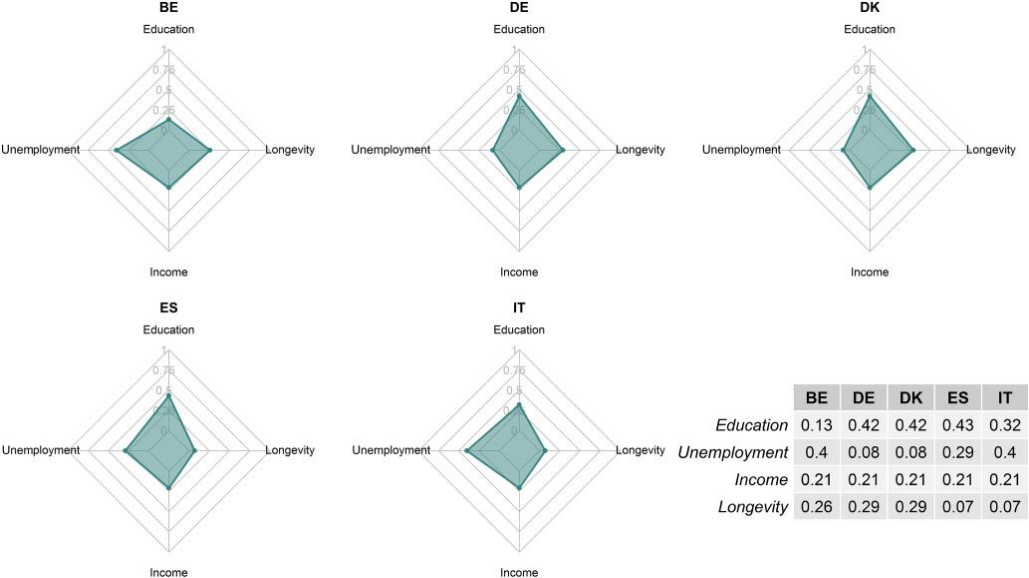
Direct effect on social inclusion Index given an increment in each criterion.

Our results are shown graphically in Figure 9, where each net reports the coefficient corresponding to the increase in Social Inclusion following a unitary improvement in the specific indicator, by country. Figure 9 also includes a table where the coefficients are explicitly outlined. Because of the non-additive nature of the experts’ preferences, and because countries baseline performances are rather different, the direct effect of each indicator on Social Inclusion differs across countries. We believe this is a small but informative additional contribution of our analysis, as it shows that policy interventions need to consider what areas of Social Inclusion could, based on experts’ evaluations, lead to the largest gains in the overall societal welfare (measured, in this case, with the Social Inclusion Index). Moreover, our results show that a top-down approach which focus on improving the same dimension in all areas might have large differential effects, depending on the starting levels of performance in that area. In general, our results show that each country would benefit from an improvement in the indicators where the current performance is worse. This explains why, for example, improvements in longevity (unemployment) lead to much smaller (larger) gains in Italy and Spain than in Denmark and Germany: longevity (unemployment) levels are much higher (lower) in Italy and Spain than they are in Denmark and Germany to begin with.

We believe this analysis should be expanded in at least two ways. First, it would be important to estimate the indirect effects that any specific improvement in a given indicator would have on the overall index. Second, future work should attempt at evaluating the monetary effectiveness of improving Social Inclusion and comparing it against the costs of implementing the policies needed to produce such improvement.

### 4.4 Comparability with other measures of Social Inclusion

In this section, we discuss our findings in light of previous studies which analysed Social Inclusion in Europe (and beyond) and emphasise the additional contribution of our methodology. We will limit the discussion to the countries which are included in our study.

Our results are in line with those emerging from two broad measures of social welfare such as the EU’s At-Risk-Of-Poverty-and-social-Exclusion (AROPE) index and the UN’s Human Development Index (HDI, see Section 2 for a definition). We graphically summarise the results from AROPE and HDI in the online supplementary material, Appendix 1.8. In the HDI study, the Mediterranean countries have very similar scores, and lower than the Continental countries by around 0.1 points (10% of the total range of the HDI) in the 1990s, while this gap has narrowed to 0.05 points in more recent years. The HDI profiles of Belgium, Denmark and Germany are very close throughout the years. The HDI index has been steadily increasing from 1990s to 2010, and then became flatter in the last decade, somewhat signalling an effect of the Great Recession. As per the AROPE index, which is available since 2015, all countries see a constant reduction in the percentage of population at risk. Mediterranean countries always show a higher prevalence of social exclusion (between 25% and 30%), although Italy’s progress seems more virtuous than Spain. Continental countries range between 17% and 22%.

Conversely, our results contrast with the picture emerging from other recent studies employing data-driven techniques to generate measures of Social Inclusion using very similar (or exactly the same) indicators that we use. Rogge & Konttinen (2018) develop an index of Social Inclusion through the Benefit-of-the-Doubt method. In their study, Italy ranks as second highest performer out of 29 countries in 2015, ahead of Denmark (7th), Germany (19th) Belgium (23rd) and Spain (29th). Lefebvre et al., (2010) employ a similar method as the previous study and produce a ranking of Social Inclusion where Spain is 1st alongside Denmark, while Germany is 12th, Belgium 13th and Italy 14th. Both studies suggest that Mediterranean countries can be regarded as highly efficient, relative to the observed best practice. A similar finding emerges from the analysis of Carrino (2016), which shows that Italy’s performance in Social Inclusion is as good as the other continental countries (while Spain performs notably worse), and improving, until the beginning of the 2010s.

The inconsistencies between the general findings of our work and the aforementioned works relies in the weighting systems adopted and the normalisation procedure. For example, in the study by Rogge & Konttinen (2018), Italy’s score is computed by assigning 85% of the weight to the education indicator (which is Italy’s relative best indicator among those considered in the study, which excludes the longevity indicator), while Germany’s and Denmark’s are assigned 66% of the weight to the poverty indicator, 23% to education, and 5% to the remaining indicators. In the study by Lefebvre et al., (2010), the weight assigned to longevity is, on average 17%, with 46% assigned to education, 24% to unemployment. In the data-driven study by Carrino (2016), longevity carries 55% of the weight, labour market 25%, education and poverty 10% each. Moreover, the data are normalised based on a data-driven method (while in our paper the normalisation is informed by experts-elicited thresholds), which changes the interpretation of the units of measurement, as discussed in Section 3.3.3.

**Figure 10.**
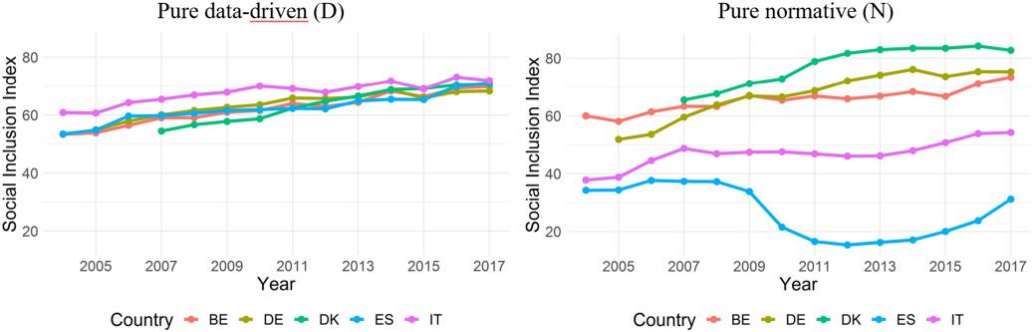
Social inclusion indices from two different approaches, pure data-driven and pure normative. Model D (pure data-driven) employs the PCA as aggregation method, and a data-driven min–max function as normalisation method; model N (pure normative) employs expert-based Choquet integral as aggregation method, and an expert-based min-max as normalisation method. Hence, the values of the Social Inclusion index in model D are not comparable to the values in model N, due to the different normalisation adopted.

Taken at face values, this comparison seems to suggest that a stark contrast with the weights derived from the preferences of our policy-experts (Section 4.1). However, we argue that attempting to compare data-driven and normative indices requires recalling that the underlying methodological rationales are very different, which reflects into an inherently different nature of the interpretations of the results. To show this, we have re-estimated our Social Inclusion index by adopting a data-driven approach as in Carrino (2016), where we use the Principal Component Analysis as an aggregation function. We hereby summarise our findings, while a more detailed description of methods and results is available in online supplementary material, Appendix 1.8. We have applied the PCA on a set of indicators normalised through a data-driven min–max function (as in, e.g. Döpke et al., (2017) and Peiró-Palomino (2018)). We call to this model as pure Data-driven (D). In our PCA analysis, 41% of the weight is assigned to longevity, while around 20% of the weight goes to each of the remaining dimension. Unsurprisingly, results from model D (shown in the left panel of Figure 10) are consistent with the results of other studies adopting data-driven techniques (Carrino 2016; Lefebvre et al., 2010; Rogge & Konttinen 2018). They show that countries have similar levels of Social Inclusion, and generally improving over time, and that Mediterranean countries are among the top performers throughout. This result is very different from that emerging from our main model, a normative model (we call it model N, in what follows), which are also reported for convenience in Figure 10.

We argue that the policy conclusions that could be taken from model D are different from those that could emerge from the results of model N). Model N relies on assumptions that are explicitly linked to value judgements and should be considered as an informative complementary evidence to the findings of model D, which is instead grounded on statistical assumptions. As Döpke et al. (2017) put it, in the PCA method the dimension weights are determined based on proportions of explained variances, yet ‘there is no reason to suppose that a statistical property, such as the correlation between dimensions, captures meaningful trade-offs between these dimensions with respect to well-being’. Stated otherwise, the weights underlying the PCA analysis are ‘not a measure of the theoretical importance of the associated indicator’ (Giovannini et al., 2008). A similar reasoning could be drawn for the data-driven methods applied in the aforementioned papers.

To conclude, comparing data-driven and normative indices requires, in the words of Decancq & Lugo (2013), to distinguish between descriptive statements (about what is) and normative statements (about what ought to be). They refer to works of Popper (1948) and Hume (1739), highlighting that we should not automatically ‘derive a statement about values from a statement about facts’. The data-driven methods lead to statements about facts, while the normative-driven methods lead to statements about values. Yet, more in general, our view is that normative methods should complement (not substitute for) the evidence already compiled from data-driven methods. In particular, normative methods complement data-driven methods in that they provide indices where the weights have an explicit economic interpretation, and the outcome carries a statement of values, provided that the sample of experts is clearly characterised and informative (see Section 3.3.1).

Sometimes, the two approaches might coincide. In the specific case of social inclusion, the two approaches lead to markedly different results. The data-driven ‘positive’ interpretation of the observed data suggests that, on average, countries performances in the four sub-dimensions of social inclusion balance out: Italy and Spain outperform the other countries in some dimensions, yet are out-performed in other dimensions, so that the overall level of performance is roughly similar across countries. The survey-driven ‘normative’ interpretation complements this view by highlighting how, under a specific set of social preferences elicited from policy experts, Italy and Spain exhibit weaknesses in several dimensions of social inclusion, which lead to a substantially worse condition of social inclusion with respect to other countries; such weaknesses are not fully compensated by the stronger performance that Italy and Spain exhibit, with respect to other countries, in other dimensions.

The contrast between the two results is entirely explained by their different conceptual approaches: as we will argue in the Conclusions to this paper, no method is objective per se. Yet, we believe that presenting both sets of results can provide the policymaker with a richer and more informed ground upon which policy choices can be made.

## 5. Discussion

While Social Inclusion has become central to the policy debate within the European Union, there is limited comparative evidence on how countries have performed in reducing Social Inclusion in the last decades. While some recent studies have provided multidimensional indices of Social Inclusion for Europe, their results do not aim to provide a normative evaluation of the observed levels of Inclusion across countries (Decancq & Lugo 2013).

This paper introduced a rigorous approach for the construction of a normative-based index of Social Inclusion, using European regional longitudinal data from Eurostat.

We believe our work leads to several important contributions which have large policy relevance. First, we use a non-additive method to aggregate sub-dimensions, the Choquet Integral, which allows to separately model the relationships between each pair of sub-dimensions, e.g. their degree of complementary or substitutability. This allows us to improve on methods which consider all outcomes as independently related to Social Inclusion (e.g. the Social Protection Committee report, which defines Social Inclusion based on the share of population which exhibits either a high poverty rate, or a high material deprivation rate, or high prevalence of (quasi)jobless households). However, our method also augments non-linear approaches, such as the geometric aggregation models, which assume that the elasticity of substitution between outcomes is the same, for all pairs of outcomes. For example, our model allows for combined shortcomings in education and labour market outcomes to affect Social Inclusion differently than combined shortcomings in education and health. The importance of evaluating synergies and redundancies between sub-dimensions of Social Inclusion can prove very valuable to policymakers, as it suggests that social policies should not be designed in ‘institutional silos’, but rather with a broad perspective that acknowledges that outcomes from different social areas (e.g. labour market, education, health) interact in affecting population welfare. Moreover, our study shows that improvements in a given indicator might lead to very different effects on the overall level of Social Inclusion, depending on the initial performances exhibited by each country. Overall, improving the dimensions that exhibit poor levels of performance would lead to higher gains compared to improving a dimension that is already at a high level of performance.

Second, we elicited the parameters of the aggregation function from a population of expert decision-makers in social policies in Italy. Such experts’ preferences represent, although subjectively, a policy-perspective on actual needs, and therefore might reflect in very different weights than those derived with data-driven methods. Moreover, the data have been standardised based on judgment thresholds set a priori and independently by academic experts in Economics, rather than employing common normalisation techniques based on observed data. Hence, our index has a clear normative interpretation, and represents a direct evaluation of the overall performance of a region or country. Moreover, our methodological approach is potentially applicable to most conceptual frameworks related to multifaceted indices, e.g. indices of transparency (Galli et al., 2017), active ageing (Floridi & Lauderdale 2022), or well-being (Peiró-Palomino 2018).

We also note that the commission which introduced the conceptual framework of Social Inclusion (see Atkinson et al., (2002)) stressed that Social Inclusion should be evaluated by looking at performance achieved, rather than at nations’ or regions’ ranking, while Atkinson et al., (2004) highlighted that the raw indicators on which the Social Inclusion index is based should carry a strong normative interpretation. We believe that, for all the aforementioned reasons, our Social Inclusion index offers a valuable and policy-relevant contribution. Our normative method offers a strong policy perspective, as it is grounded in the preferences expressed by experts in both its aggregation and its normalisation stage.

Our results highlight an important divide in Social Inclusion between Mediterranean countries such as Italy and Spain, and Northern/Central countries such as Denmark, Germany and Belgium. On a scale from 0 (very low and socially undesirable Social Inclusion level) to 100 (very high and socially desirable Social Inclusion level), the former countries’ Inclusion index ranges between 15 and 50, between 2004 and 2017). Among the latter group, the index ranges between 50 and 80. The spread between the two groups has significantly increased after the recent financial crisis, with Italy on a stagnating path and Spain’s index dropping from a score of around 40 (pre-crisis) to 15 (post-crisis). Moreover, we showed that regional inequalities in Social Inclusion vary widely across countries. On the one hand, Italy’s regional context has grown increasingly unequal over time, with some regions faring extremely well and other extremely bad in the Social Inclusion index. Conversely, Germany and Denmark show rather small regional variation, and a narrowing trend over time. Our results, therefore, suggest that the recent decades have not seen a reduction in disparities across and within countries, and highlight the existence of areas facing levels of Inclusion which are highly undesirable. This highlights the need for Social Inclusion policies that could help reduce the gaps within countries, and increase Social Inclusion in Mediterranean countries to reduce the gap with other Continental countries.

These results can be read in light of the recent reports by the European Social Protection Committee (2018), which focus on the monetary and labour-market dimensions of Social Inclusion. The report has documented a persistent divergence across Member States with respect to material deprivation and risk of poverty. Overall, our results provide a somewhat more worrisome picture, as our index also includes health and education outcomes, and hence is able to better represent the complexity of dimensions which underly Social Inclusion. Our findings are in line with evidence from alternative conceptual frameworks of Social Inclusion such as the Human Development Index and the European Union AROPE index (UNECE 2022). Conversely, our results challenge recent evidence based on the same conceptual framework we employ, but which adopts data-driven approaches e.g. Rogge & Konttinen (2018) and Carrino (2016), who highlighted high and improving trends in Social Inclusion for Mediterranean countries.

However, our approach is not free from limitations. First, given that we focused on administrative regions, our country selection is limited by data availability, as we could not include countries which only had data for statistical regions, or lacked several years of data for administrative regions. However, further analyses could enlarge the sample size, for example, by focusing on countries rather than on regions.

Moreover, our method, as arguably any method for multidimensional welfare evaluation, is not objective per se. For example, the index parameters as well as the results could change with the choice of the expert panel. However, since methodological ‘objectiveness’ in building a multidimension indicator of a latent abstract construct is at odds with the inherent subjectivity of such construct, several calls have been made for strategies that enhance transparency (Sen & Anand 1997) and expressiveness of the methods (Decancq & Lugo 2013; Hüllermeier & Schmitt 2014). Moreover, our approach could be applied to a larger scale, e.g. by engaging with a European or Global panel of experts, in order to build an overarching normative policy instrument for monitoring Social Inclusion.

Finally, our operationalisation of Social Inclusion, although in line with several previous studies on the topic, could be challenged. Alternative empirical models could be proposed to measure the latent phenomenon, with a larger number of sub-dimensions (UNECE 2022). While an increase in the number of attributes could prove cumbersome for the experts involved in the scenario evaluation process, recent studies have shown that a complex conceptual model can be operationalised in a *tree-shaped* (nested) structure, where each node of the tree is constituted by a limited number of attributes (Bertin et al., 2018).

## Supplementary Material

qnad106_Supplementary_Data

## Data Availability

The data in this study are openly available from the EUROSTAT website, at https://ec.europa.eu/eurostat/web/regions/data/database.

## Footnotes

The list of the 10 primary indicators includes, by sub-dimension: Income: Poverty rate (after social transfers), Persistent risk-of-poverty rate, Relative median at risk-of-poverty gap, Inequality of income distribution; Labour market: Long-term unemployment, Regional cohesion, Persons living in jobless households; Education: early school leavers; Health: life expectancy at birth, Self-defined health status by income level.

Regional data for other countries (e.g., Greece, France, Czech Republic, and Norway) were either not available, or only available for statistical regions (not administrative regions).

A partial exception would be the nested CES framework proposed by F. Bourguignon and S. R. Chakravarty (2003). The measurement of multidimensional poverty. *The Journal of Economic Inequality,* 25–49, which allows for different degrees of sustainability for different (exclusive) subgroups of attributes.

The reduced or *k* order-additive model (Grabisch, 1997), which considers interactions only between subset of limited cardinality (order *k*), can be applied to reduce the numerical complexity of the problem.

As the name of the position differs across regions, we compared regions’ organisation chart, and verified that the specifications of the experts’ roles be similar across regions. For example, in the Campania Region, the role is called “Director of Operative Unit for Welfare and Equal Opportunities” (Unità Operativa Dirigenziale Welfare dei servizi e pari opportunità). Its duties involve the “research, planning and coordination of Social policies for family, maternity, youths, older people; definition and implementation of policies on poverty, disability, and cognitive impairment”.

Experts were first contacted via email, then through a phone-call and, upon acceptance, a face-to-face meeting was scheduled. The fieldwork took place between 2014 and 2015. Out of 20 DGSP, 12 accepted to take part to the survey: these are the DGSP from Abruzzo, Alto-Adige, Campania, Friuli-Venezia Giulia, Emilia-Romagna, Liguria, Lombardia, Marche, Piemonte, Puglia, Toscana, Veneto. Refusals to take part to the survey were always justified with lack of available time.

Alternative cardinal methods include, e.g., the Maximum-split, the Minimum variance, the Minimum distance. In high-dimension problems, if setting specific values in the fuzzy contest proves to be cumbersome for the expert, an ordinal-based approach can be employed, in which the expert determines ordinal preferences among alternatives/scenarios. We refer the reader to Grabisch et al. (2008) for a review.

Out of the 3^4^ = 81 possible scenarios (i.e., any possible permutation), which would require an unrealistic effort from the DM, we implemented the fractional factorial technique to obtain the full-rank matrix with 27 scenarios (available in the online supplementary material, Appendix 1.3).

For example, a scenario which conveyed a mid-high level of Inclusion would need to be dragged close to the trivial-scenario “7.5”.
