# Supplementary material for: Measuring Social Inclusion in Europe: a non-additive approach with the expert-preferences of public policy planners
Source: J R Stat Soc Ser A Stat Soc. 2023 Sep 5;187(1):231–59. doi: 10.1093/jrsssa/qnad106 (PMC10782991; doi:10.1093/jrsssa/qnad106)
Supplement: qnad106_Supplementary_Data [file qnad106_supplementary_data.docx]

# Appendix

## Descriptive statistics: time trends for the four indicators

Figure 11, time series data of the criteria used in the composite index


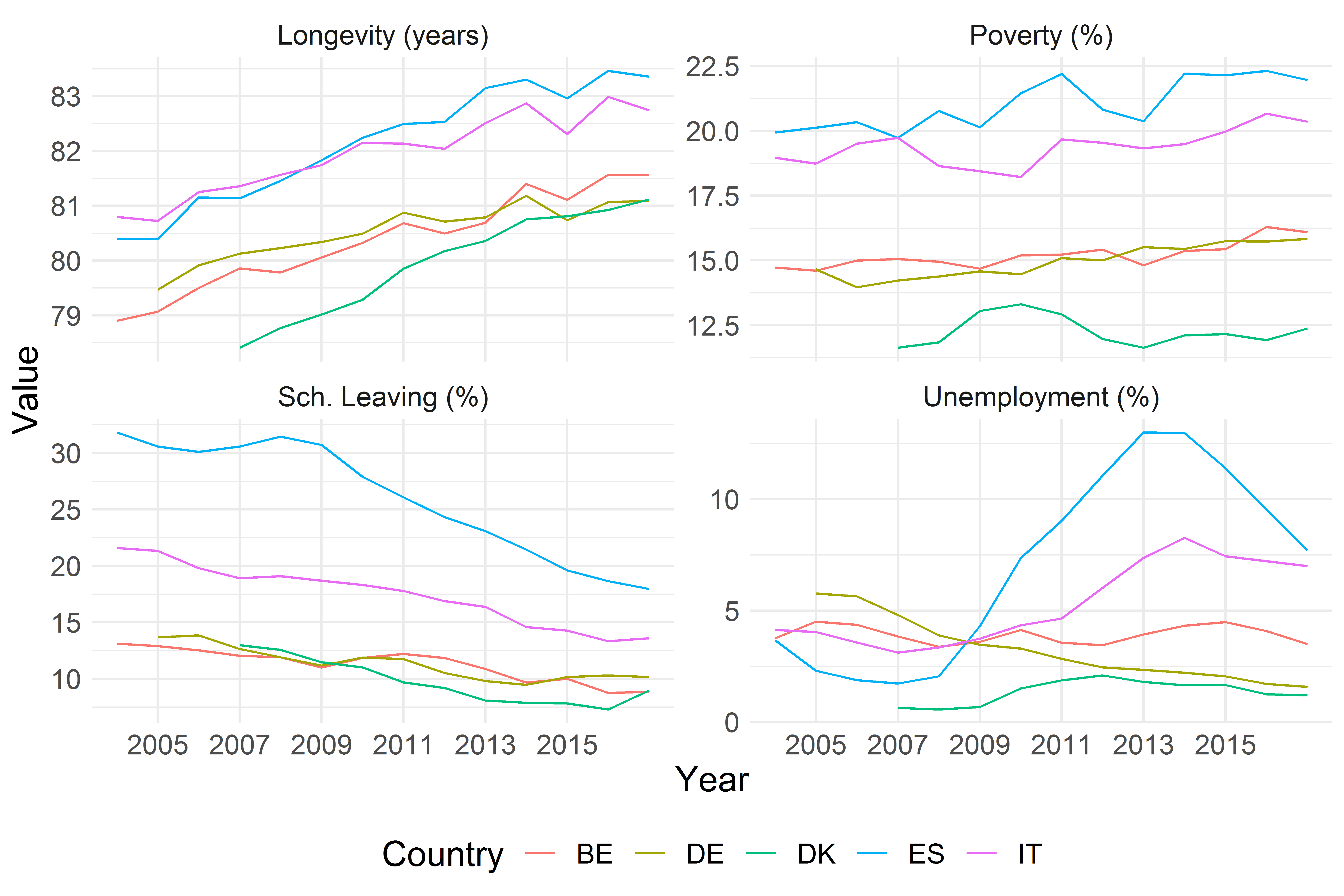


## Details on the normalisation strategy

In this paper we adopt the *min-max normalisation function* (Giovannini, Nardo, Saisana, Saltelli, Tarantola and Hoffman 2008), with expert-driven thresholds.

For each attribute *x* observed in region *i* at a time *t* (we drop the previously used attribute-specific *j* index to simplify the notation), the corresponding normalised value is defined as:

|  | $\nu_{MM+}^{i,t}\left( x_{+}^{i,t} \right)=100*\frac{x^{i,t}-b_{+}\min\left( x_{+} \right)}{b_{+}\max\left( x_{+} \right)-b_{+}\min\left( x_{+} \right)}$ $\nu_{MM+}^{i,t}\left( x_{+}^{i,t} \right)=0\text{ if }x_{+}^{i,t}\leq b_{+}\min\left( x_{+} \right)$ $\nu_{MM+}^{i,t}\left( x_{+}^{i,t} \right)=100\text{ if }x_{+}^{i,t}\geq b_{+}\max\left( x_{+} \right)$ | $\nu_{-}^{i,t}\left( x^{i,t} \right)=100*\frac{b_{-}\max\left( x \right)-x^{i,t}}{b_{-}\max\left( x \right)-b_{-}\min\left( x \right)}$ $\nu_{-}^{i,t}\left( x_{-}^{i,t} \right)=0\text{ if }x_{-}^{i,t}\geq b_{-}\max\left( x_{-} \right)$ $\nu_{-}^{i,t}\left( x_{-}^{i,t} \right)=100\text{ if }x_{-}^{i,t}\leq b_{-}\min\left( x_{-} \right)$ |
| --- | --- | --- |

where *ν_+_* is used when *x* has positive polarity (i.e., it is a “good”) and *ν_-_* is used when *x* has negative polarity (i.e., it is a “bad”). The coefficients b*min_i_* and b*max_i_* are the highest and lowest values to be used as benchmarks for the *x* variable for region *i*. When *x* has positive polarity, b_+_max corresponds to a more desirable performance than b_+_min, while the opposite is true when *x* has negative polarity. The *min-max* strategy rescales indicators into an identical range [0,100]. A constant normalised value is assigned to indicators exceeding the benchmarks, while indicators which fare within these benchmarks are proportionally converted into the 0-100 scale. Hence, *ν* is stepwise continuous.

The benchmarks of the normalisation function can either reflect some statistical properties of the data (e.g., the *data-driven* strategy), or have a normative connotation stemming from the preferences elicited from some stakeholders group, e.g., field-experts, members of institutions, citizens (Kim, Kee and Lee (2015) and Decancq and Lugo (2013) produce a recent review of elicitation strategies in the context of the aggregation function).

If the parameters were data-driven, then the normalised variables would be suitable to be interpreted under a statistical perspective, free from a normative connotation, yet it would be harder to determine what did they reflect in economic terms (Lefebvre, Coelli and Pestieau 2010). As an example, in the data-driven min-max, a variable with transformed-value equal to “0” just implies it being “the last one”, or “the worst one” observed among the available data, which does not necessarily corresponds to an undesirable condition of Well-being.

An alternative to the data-driven normalisation would require to incorporate some value judgments in the normalisation. This translates to linking the extreme values “0” and the “100” with, e.g., a certain definition of desirability, thus making the normalisation independent from the data. When an indicator lies above or below such fixed “goalposts”, further variations do not contribute to the composite measure (see e.g., the discussion in Anand and Sen (1994), Klugman, Rodríguez and Choi (2011), Ravallion (2012), Lefebvre, Coelli and Pestieau (2010), Gidwitz et al. (2010) and Mazziotta and Pareto (2015)). As an example, the Human Development Index adopts goalposts in terms of objective upper and lower bounds (“subsistence” minimum or “satiation” points).

In this paper, we employ the thresholds elicited by Carrino (2016) by means of an online survey among 149 academics and researchers from the Department of Economics and Management at the Ca’ Foscari University of Venezia, Italy (details on the methodology are available in the aforementioned paper). The survey elicited, for each social-inclusion indicator, a positive threshold corresponding to a “certainly desirable and favourable condition of Well-being” (normalised value of 100), as well as a negative threshold for a “certainly undesirable and harmful condition of Well-being” (normalised value of 0).

The selected benchmarks correspond to the median answers to the survey, as summarised in the following Table.

Table 4, survey-elicited benchmarks for normalisation (interquartile range in parenthesis)

|  | Median elicited minimum  (25p – 75p) | Median elicited maximum  (25p – 75p) |
| --- | --- | --- |
| Longevity | 73 years (70 – 75) | 83 years (80 – 85) |
| Early school leaving | 10% (5 – 10) | 20% (15 – 25) |
| Long-term unemployment | 3% (2 – 4) | 9% (5.25 – 10) |
| At-risk-of-poverty rate | 5% (3 – 7) | 20% (17 – 21.5) |

Note: details on the survey’s results are available in Carrino (2016), Carrino (2017).

## List of scenarios

Each of the four Social Inclusion dimensions (longevity, long-term unemployment, poverty rate, school dropouts) can take three levels, i.e., High (10), Intermediate (5) or Low (0). The list of scenarios is as follows:

Table 5, List of Scenario included in the elicitation

| Scenario | Education | Employment | Income | Health |
| --- | --- | --- | --- | --- |
| 1 | 100 | 100 | 100 | 100 |
| 2 | 100 | 50 | 50 | 100 |
| 3 | 100 | 0 | 0 | 100 |
| 4 | 50 | 100 | 50 | 100 |
| 5 | 50 | 50 | 0 | 100 |
| 6 | 50 | 100 | 0 | 50 |
| 7 | 0 | 100 | 0 | 100 |
| 8 | 100 | 100 | 50 | 50 |
| 9 | 0 | 0 | 50 | 100 |
| 10 | 50 | 0 | 100 | 100 |
| 11 | 0 | 50 | 100 | 100 |
| 12 | 100 | 0 | 100 | 50 |
| 13 | 100 | 50 | 0 | 50 |
| 14 | 50 | 50 | 100 | 50 |
| 15 | 50 | 0 | 50 | 50 |
| 16 | 0 | 100 | 100 | 50 |
| 17 | 0 | 50 | 50 | 50 |
| 18 | 0 | 0 | 0 | 50 |
| 19 | 100 | 100 | 0 | 0 |
| 20 | 100 | 50 | 100 | 0 |
| 21 | 100 | 0 | 50 | 0 |
| 22 | 50 | 100 | 100 | 0 |
| 23 | 50 | 50 | 50 | 0 |
| 24 | 50 | 0 | 0 | 0 |
| 25 | 0 | 100 | 50 | 0 |
| 26 | 0 | 50 | 0 | 0 |
| 27 | 0 | 0 | 100 | 0 |

## Full results from the experts’ elicitation process

Table 6, estimated Choquet parameters from decision makers' preferences elicitation

|  | Shapley values | | | | Interactions | | | | | | Orness | R^2^ |
| --- | --- | --- | --- | --- | --- | --- | --- | --- | --- | --- | --- | --- |
|  | Ed | Un | Po | Le | Ed  & Un | Ed  & Po | Ed  & Le | Un  & Po | Un  & Le | Po  & Le |  |  |
| dm1 | 0.18 | 0.35 | 0.32 | 0.15 | 0.09 | -0.09 | 0.22 | -0.34 | 0.10 | -0.15 | 0.58 | 0.88 |
| dm2 | 0.32 | 0.19 | 0.28 | 0.21 | 0.21 | -0.05 | 0.09 | -0.06 | 0.01 | 0.07 | 0.42 | 0.74 |
| dm3 | 0.36 | 0.21 | 0.17 | 0.26 | 0.11 | 0.18 | 0.17 | -0.08 | 0.27 | 0.05 | 0.30 | 0.79 |
| dm4 | 0.32 | 0.26 | 0.20 | 0.22 | 0.19 | 0.09 | 0.18 | -0.03 | 0.18 | -0.15 | 0.43 | 0.72 |
| dm5 | 0.31 | 0.17 | 0.24 | 0.28 | 0.02 | -0.01 | 0.01 | -0.04 | 0.02 | 0.10 | 0.44 | 0.67 |
| dm6 | 0.13 | 0.16 | 0.35 | 0.35 | 0.14 | 0.12 | -0.05 | -0.04 | 0.15 | 0.13 | 0.41 | 0.77 |
| dm7 | 0.48 | 0.22 | 0.16 | 0.14 | 0.10 | 0.03 | 0.15 | 0.05 | 0.01 | 0.08 | 0.41 | 0.80 |
| dm8 | 0.40 | 0.17 | 0.26 | 0.16 | 0.03 | -0.04 | 0.03 | -0.05 | 0.05 | -0.05 | 0.50 | 0.61 |
| dm9 | 0.28 | 0.20 | 0.34 | 0.17 | 0.07 | -0.26 | 0.28 | 0.04 | -0.10 | -0.18 | 0.54 | 0.78 |
| dm10 | 0.24 | 0.25 | 0.31 | 0.20 | 0.03 | 0.24 | 0.29 | -0.03 | 0.07 | 0.21 | 0.30 | 0.80 |
| dm11 | 0.30 | 0.32 | 0.23 | 0.15 | 0.26 | 0.07 | 0.19 | 0.15 | 0.20 | 0.00 | 0.29 | 0.82 |
| dm12 | 0.34 | 0.23 | 0.20 | 0.23 | 0.06 | 0.07 | 0.11 | 0.12 | 0.17 | -0.03 | 0.38 | 0.85 |
| Dm  fusion | 0.31 | 0.23 | 0.26 | 0.21 | 0.11 | 0.03 | 0.14 | -0.03 | 0.09 | 0.01 | 0.42 |  |

Note: Ed=Education; Un=Unemployment; Po=poverty; Le=Life Expectancy

## Details on capacity elicitation method

We formulate the quadratic optimization problem to retrieve the Choquet capacities for a generic expert involved in the elicitation. in the simplified case of a 2-additive Choquet model. This choice is made in the interest of clarity, with no substantial difference from the more general case, following Bertin, Carrino and Giove (2018). The problem can be expressed as:

| 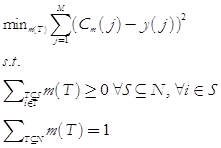 | (15) |
| --- | --- |

where $C_{m}\left( j \right), j=1,..,M$ are the value of the Choquet integral for the $j$-th computed using the 2-order model. The unknown variables are the values of the second order non additive measure $m\left( T \right), T\subseteq N, card (T)\leq2$, and the problem is quadratic given that the Choquet integral $C_{m}\left( j \right)$ is linear w.r.t. measure values. This formulation is very general, and the constraints cardinality increases exponentially with $n$, making more difficult even a numerical solution. But limiting to a second order model, having in mind that $(T)=0$ $\forall T\subseteq N, card \left( T \right)>2$, the complexity is strongly reduced, and the quadratic optimization problem can be easily solved by standard techniques, at least until $n$ remains inside acceptable values.

Let us offer a simple applied example of the methodology used to identify the Möbius coefficients for a node consisting of two criteria (A, B), for which the scenarios 1a and 2a of Table 6 are submitted to the experts for an evaluation, under a 2-additive Choquet framework. Each criterion can take values from 0 to 1. Let us assume that an expert evaluates the first scenario with value 0.38, and the second with value 0.75.

The minimisation problem can be written as follows:

| 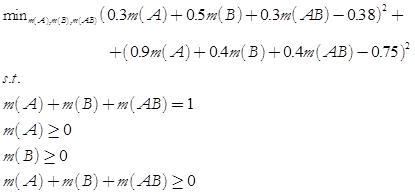 | (16) |
| --- | --- |

Where the last constraint is implicit in the first. The problem’s solution gives *m(A)=0.7*, *m(B)=0.4*, *m(AB)=-0.1 .*

Table 7, scenario examples

| Scenario no. | Fictional value for criterion A | Fictional value for criterion B | Scenario-evaluation by the expert |
| --- | --- | --- | --- |
| 1a | 0.3 | 0.5 | 0.38 |
| 2a | 0.9 | 0.4 | 0.75 |
| 1b | 0.3 | 0.6 | 0.3 |
| 2b | 0.5 | 0.8 | 0.5 |

## Summary performance of countries in 2017

Table 8 reports the observed performance in the four statistical indicators representing the dimensions of Social Inclusion, in the year 2017.

Table 8, Country performance in 2017

| Country | *Education* | *Employment* | *Income* | *Longevity* |
| --- | --- | --- | --- | --- |
| *BE* | 95.3 | 77.3 | 39.0 | 85.6 |
| *DE* | 90.6 | 99.6 | 28.1 | 80.9 |
| *DK* | 96.8 | 100.0 | 50.8 | 81.1 |
| *ES* | 29.1 | 30.8 | 16.5 | 97.8 |
| *IT* | 63.5 | 54.0 | 27.9 | 96.6 |

## Social Inclusion index with alternative fusion expert preferences

The following figure shows the trend and levels of our Social Inclusion index computed based on an alternative aggregation of our experts’ preferences. Specifically, rather than following the methodology described in section 3.4, we computed the simple average of the experts’ preferences. The results are almost identical to our main findings.

Figure XII, Social Inclusion index computed based on the average of experts’ preferences


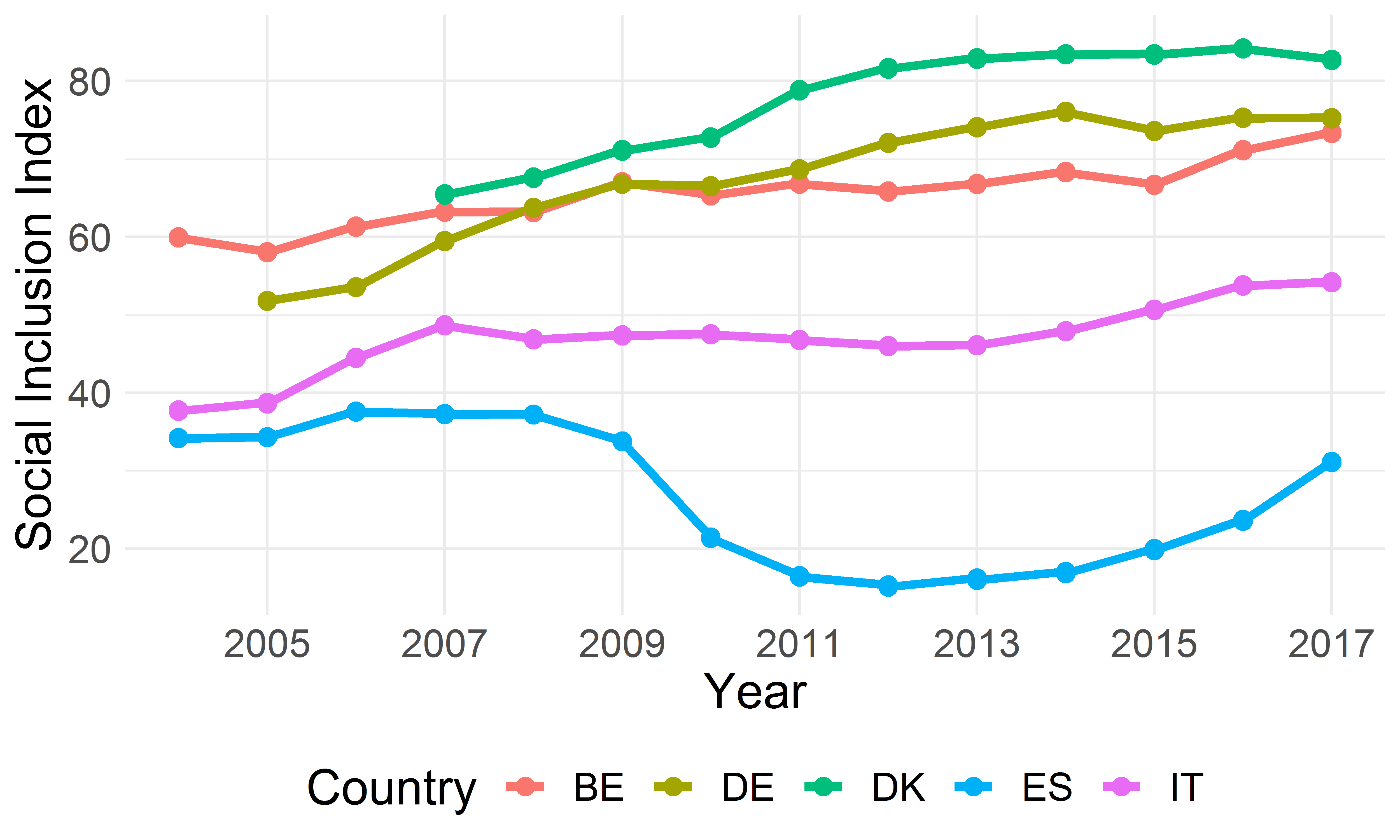


## Full results from comparability analysis

*Comparison with Human Development Index, and At-Risk-Of-Poverty-and-social-Exclusion index*

Figure 13, Human Development Index scores


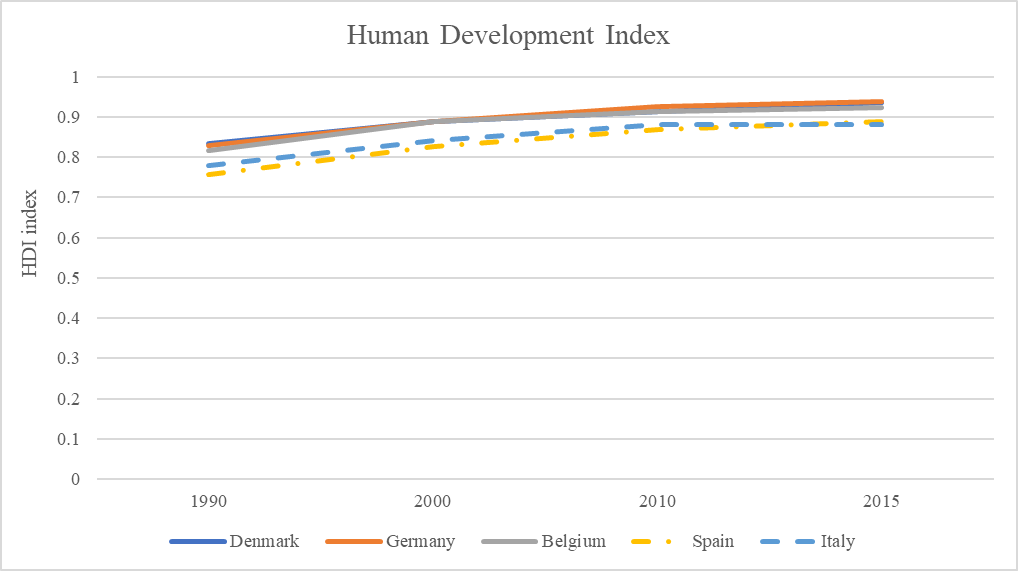


Note: data extracted from the United Nations Development programme at https://hdr.undp.org/data-center/documentation-and-downloads

Figure 14, At Risk Of Poverty and Social Exclusion (AROPE)


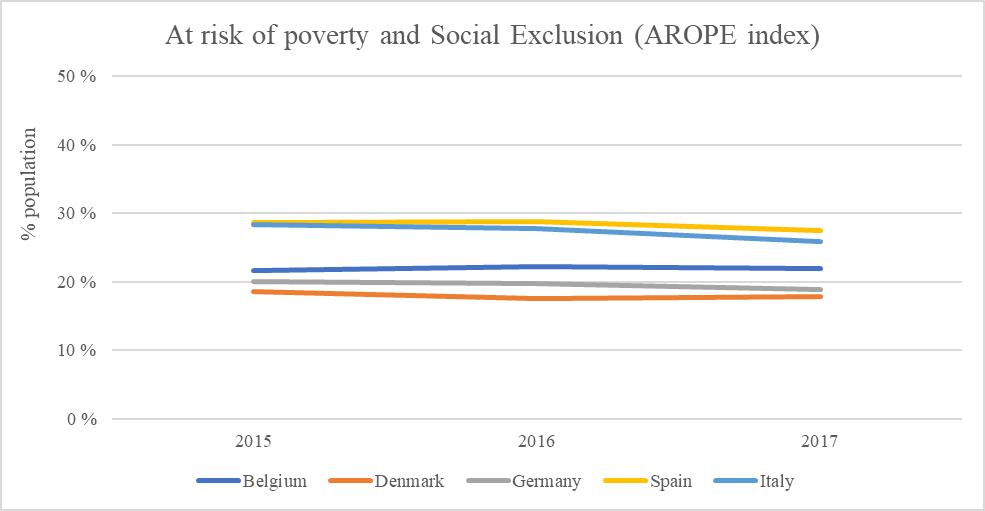


Note: data extracted from the Eurostat Living Conditions in Europe database at:

<https://ec.europa.eu/eurostat/statistics-explained/index.php?title=Living_conditions_in_Europe_-_poverty_and_social_exclusion>

*Comparison with results based on PCA-based aggregation*

We have re-estimated our index using the Principal Component Analysis (PCA) as an aggregation function. We have applied the PCA on the set of indicators normalized through a data-driven min-max function (as in, e.g., Döpke, Knabe, Lang and Maschke (2017) Carrino (2016) and Peiró-Palomino (2018)). We call this the pure Data-driven model (D).

We adopted Farnia (2019) approach for criteria weighting by means of Spectral Value Decomposition, choosing 2017 as baseline year for the min-max normalization. Such an approach considers the correlation of data and tends to weight proportionally more the criteria that are less correlated with others. The component is retained if its eigenvalues is greater or equal than average (greater or equal 1 with standardized data) and not on the basis of the total variance explained (usually greater or equal 80%). The final criterion weight is equal to the average of the variances explained by it in each component retained. The aim is not hence the approach that best fits the data, but the one that best fits the dimensions the data are explaining; as a consequence, in the extreme case of statistically independent data, all components are retained (because all the eigenvalues are equal one) and each criterion assume the same weight. Adopting 80% of total variance explained as a decision rule to retain components, would lead to the undesirable consequence of setting to zero the wight of 20% of the variables used in the composite index, although they are fundamental in explaining different dimensions.

According to the 2017 data, two components are retained, the first clearly represented by income, education and employment; the second by longevity; as a consequence, longevity (0.411) receives the highest weight, followed by education (0.198), income (0.199), and employment (0.190). The eigenmatrix and eigenvalues are reported in Table 7.

Table 9, Eigenmatrix and eigenvalues (year 2017) for model D

|  | *Component* | | | |
| --- | --- | --- | --- | --- |
|  | *1* | *2* | *3* | *4* |
| *Education* | -0.57 | -0.03 | 0.82 | -0.08 |
| *Employment* | -0.59 | -0.10 | -0.47 | -0.65 |
| *Income* | -0.56 | 0.32 | -0.31 | 0.69 |
| *Longevity* | 0.11 | 0.94 | 0.09 | -0.31 |
| *Eigenvalue* | *2.65* | *1.08* | *0.21* | *0.05* |

The results from the model D is shown in the following figure, alongside the baseline results from our pure normative model (N). We must recall that we cannot meaningfully compare the levels of performance between model D and model N, as the normalization is different. Nevertheless, we can comment on the differences in ranking between models D and N. Model D depicts a general positive trend in Social Inclusion, with countries having very similar levels of Inclusion throughout the period, and Mediterranean countries being among the top performers throughout. This picture is roughly consistent with the results of other studies adopting data-driven techniques (Carrino 2016, Lefebvre, Coelli and Pestieau 2010, Rogge and Konttinen 2018). The results from our main model N are strikingly different, with Italy and Spain exhibiting by far the lowest levels of Social Inclusion at any time, with an increasing gap with the performances of Continental countries, especially after the Great Recession. As we discuss in the paper (Section 4.4), the main reason for these different results relies in the weights that have been assigned to each dimension. In the PCA analysis for model D, 41% of the weight is assigned to longevity, while around 20% of the weight goes to each of the remaining dimension. The weights adopted in the pure normative model N are derived from experts preferences and are discussed in Section 4.1: education has a weight above 30%, poverty 25%, while unemployment and longevity are slightly above 20%.

Figure 15, Social inclusion indices from two different approaches, pure data-driven to pure normative.

Pure data-driven (D) Pure normative (N)


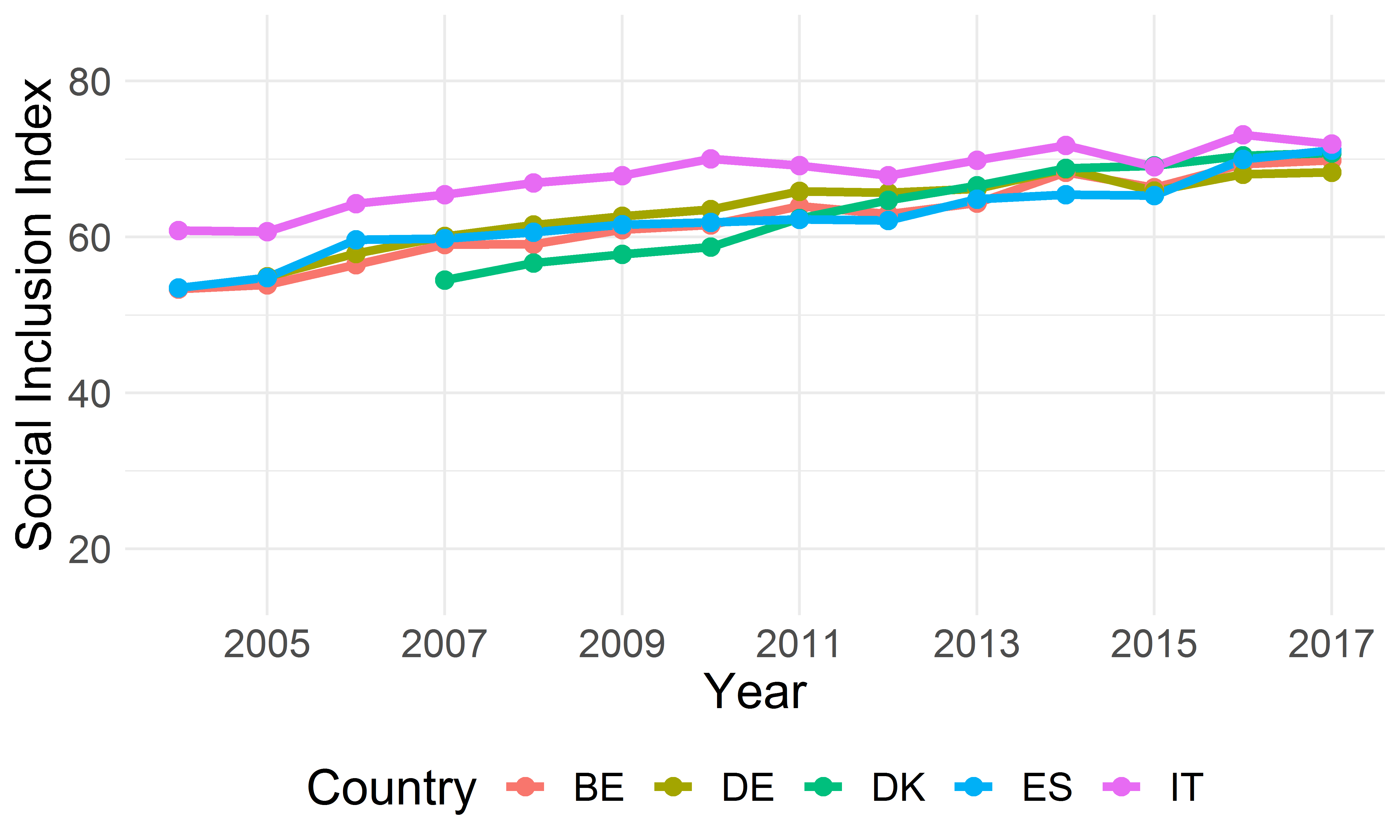

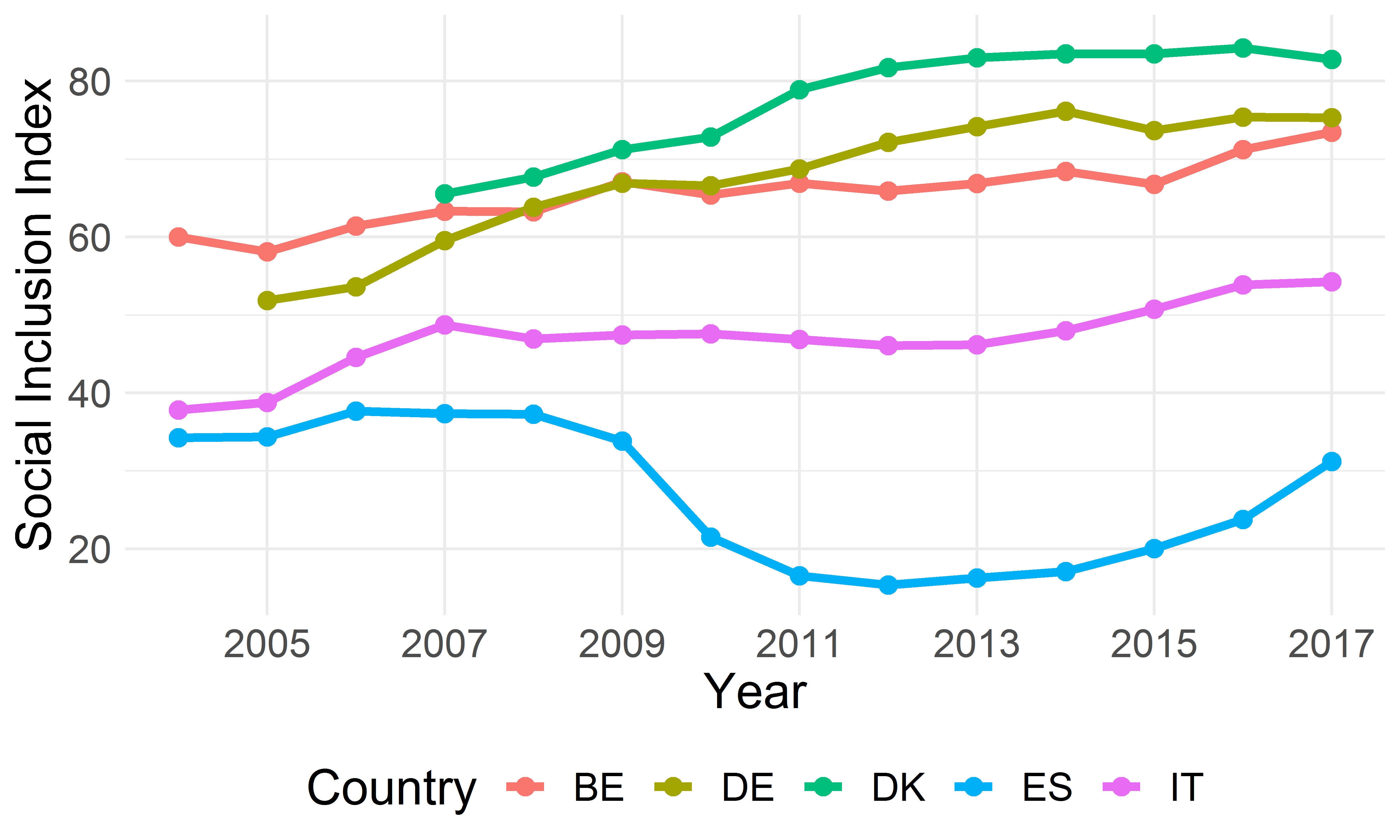


*Note: model D (pure data-driven) employs the PCA as aggregation method, and a data-driven min-max function as normalization method; model N (pure normative) employs expert-based Choquet integral as aggregation method, and an expert-based min-max as normalization method.*

*Hence, the values of the Social Inclusion index in model D are not comparable to the values in model N, due to the different normalization adopted.*

## REFERENCES

S. Anand and A. Sen (1994) Human development index: methodology and measurement: Human Development Report Office (HDRO), United Nations Development Programme (UNDP).

G. Bertin, L. Carrino and S. Giove (2018) The Italian regional well-being in a multi-expert non-additive perspective. *Social Indicators Research,* 15-51.

L. Carrino (2016) Data Versus Survey-based Normalisation in a Multidimensional Analysis of Social Inclusion. *Italian Economic Journal,* 305-345.

L. Carrino (2017) The Role of Normalisation in Building Composite Indicators. Rationale and Consequences of Different Strategies, Applied to Social Inclusion In *Complexity in Society: From Indicators Construction to their Synthesis*  (ed F. Maggino), pp. 251-289, Cham: Springer International Publishing.

K. Decancq and M. A. Lugo (2013) Weights in multidimensional indices of wellbeing: An overview. *Econometric Reviews,* 7-34.

J. Döpke, A. Knabe, C. Lang and P. Maschke (2017) Multidimensional Well-being and Regional Disparities in Europe. *JCMS: Journal of Common Market Studies,* 1026-1044.

E. Giovannini, M. Nardo, M. Saisana, A. Saltelli, A. Tarantola and A. Hoffman (2008) Handbook on constructing composite indicators: methodology and user guide.

Y. Kim, Y. Kee and S. Lee (2015) An Analysis of the Relative Importance of Components in Measuring Community Wellbeing: Perspectives of Citizens, Public Officials, and Experts. *Social Indicators Research,* 345-369.

J. Klugman, F. Rodríguez and H.-J. Choi (2011) The HDI 2010: new controversies, old critiques. *The Journal of Economic Inequality,* 249-288.

M. Lefebvre, T. Coelli and P. Pestieau (2010) On the Convergence of Social Protection Performance in the European Union. *CESifo Economic Studies,* 300-322.

M. Mazziotta and A. Pareto (2015) On a Generalized Non-compensatory Composite Index for Measuring Socio-economic Phenomena. *Social Indicators Research,* 1-21.

J. Peiró-Palomino (2018) Regional well-being in the OECD. *The Journal of Economic Inequality,* 1-24.

M. Ravallion (2012) Troubling tradeoffs in the human development index. *Journal of Development Economics,* 201-209.

N. Rogge and E. Konttinen (2018) Social Inclusion in the EU Since the Enlargement: Progress or Regress? *Social Indicators Research,* 563-584
